# Lead‐Free Cesium Manganese Halide Nanocrystals Embedded Glasses for X‐Ray Imaging

**DOI:** 10.1002/advs.202204843

**Published:** 2022-12-03

**Authors:** Kai Li, Wenchao Zhang, Luyue Niu, Ying Ye, Jing Ren, Chao Liu

**Affiliations:** ^1^ State Key Laboratory of Silicate Materials for Architectures (SMART) Wuhan University of Technology 122 Luoshi Road, Hongshan Wuhan Hubei 430070 P. R. China; ^2^ Key Laboratory of In‐fiber Integrated Optics Ministry Education of China Harbin Engineering University Harbin 150001 China

**Keywords:** cesium manganese halide semiconductor nanocrystals (NCs), glass, lead‐free, long‐term stability, X‐ray imaging

## Abstract

The toxicity of heavy‐metal Pb and instability of lead‐based halide perovskite nanomaterials are main factors to impede their practical applications in the fields of solar cells, LEDs and scintillators. In this paper, all inorganic lead‐free cesium manganese halide nanocrystals are synthesized in glass for the first time. Red photoluminescence with broad PL band, negligible self‐absorption and a high photoluminescence quantum yield of 41.8% is obtained. In addition, modulating halide component can change the Mn^2+^ ions coordination environment to obtain tunable photoluminescence from red to green. More importantly, cesium manganese halide nanocrystals embedded glasses exhibit outstanding long‐term stabilities. Theses cesium manganese halide nanocrystals embedded glasses are also highly stable against high energy irradiation and exhibit highly efficient radioluminescence, making them promising for high‐resolution X‐ray imaging. These results demonstrate that cesium manganese halide nanocrystals embedded glasses are promising eco‐friendly candidates for applications in light‐emitting diodes and scintillators.

## Graphical abstract

Lead‐free cesium manganese halide NCs embedded glasses exhibit negligible self‐absorption, long‐term stability, and high PL QY as well as excellent X‐ray scintillation performance and highly resolved X‐ray imaging.

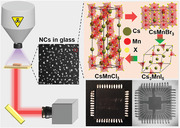

## 1 Introduction

All inorganic semiconductor nanocrystals (NCs) with high‐performance optoelectronic properties are essential components for light‐emitting diodes, lasers, photodetectors, solar cells, etc. In recent years, lead halide perovskite (CsPbX_3_, X = Cl, Br, I) NCs with excellent optoelectronic properties such as large absorption coefficient, tunable photoluminescence (PL), high photoluminescence quantum yield (PL QY), narrow full width at half maximum (FWHM), and short PL lifetime (in the order of nanoseconds), have attracted a lot of attention.^[^ ^1^, ^2^, ^3^, ^4^ ^]^ However, toxicity of heavy metal lead and poor stability of CsPbX_3_ NCs make it difficult to take full advantages of their unique optoelectronic properties. In order to improve their chemical and photo‐stabilities of CsPbX_3_ NCs, methods such as doping engineering, construction of core–shell structures, and encapsulation into organic/inorganic matrixes, have been employed to improve their stabilities against moisture, oxygen, and high energy beam irradiation.^[^ ^5^, ^6^ ^]^ Among these methods, in situ precipitation of CsPbX_3_ NCs provides one unique solution to the above‐mentioned issues, since the dense and inert glass matrix not only prevents the degradation of CsPbX_3_ NCs, but also effectively reduces the leakage of the toxic lead ions by immobilizing them in the dense glass network.^[^ ^7^, ^8^ ^]^ Poor thermal stability of CsPbX_3_ NCs is another performance limiting factor. For example, both colloidal CsPbX_3_ NCs and those precipitated in glasses also suffer from serious thermal‐induced PL quenching.^[^ ^9^, ^10^ ^]^ In addition, strong self‐absorption due to the small Stokes shift results in serious reduction in PL QY of LEDs^[^ ^7^, ^11^ ^]^ and luminous scintillators^[^ ^12^, ^13^ ^]^ based on CsPbX_3_ NCs. These features set critical bottlenecks for their practical applications, and development of lead‐free metal halide NCs with good thermal, chemical, and optoelectronic properties is still urgently needed.

Recently, lead‐free metal halides such as Ag‐based halides, Cu‐based halides, Mn‐based halides, Sn‐based halides, Sb‐based halides, and double perovskites featured by self‐trapped exciton (STE) PL, large PL band width, high PL QY, large Stokes shift, and µs‐long lifetimes, have been considered as alternatives for many optoelectronic applications.^[^ ^14^, ^15^, ^16^ ^]^ Among them, Mn‐based halides are attracting special attention since their PL properties can be tuned by changing the local coordination structure and the degree of spin‐orbit coupling of Mn^2+^ ions.^[^ ^17^ ^]^ For Cs_3_MnX_5_, Cs_2_MnX_4_, and organic‐inorganic hybrid halides, tetrahedrally coordinated Mn^2+^ ions (Mn(IV)) exhibit green PL with a narrow full width at half maximum (FWHM) of 40–60 nm and a high PLQY of 70–90%, and have been considered as environmental‐friendly green phosphors for solid‐state lighting and display devices.^[^ ^18^, ^19^ ^]^ For CsMnX_3_ halide components, octahedrally coordinated Mn^2+^ ions (Mn(VI)) usually yield red PL in the spectral range of 620–660 nm and with a FWHM of ≈100 nm.^[^ ^20^ ^]^ Besides the high PL QY, tunable PL, and large Stokes shift, these Mn‐based halides also exhibit good resistance to thermally induced PL quenching.^[^ ^21^ ^]^ These features make Mn‐based halides promising for applications in LEDs and scintillators.^[^ ^22^, ^23^ ^]^ For example, red‐LEDs made from Mn‐based halides reach a brightness of 4700 cd m^−2^ and an external quantum efficiency of 9.8%, higher than those reported from lead‐free perovskite LEDs.^[^ ^22^ ^]^ Large‐area scintillator made from TPP_2_MnBr_4_ transparent ceramic demonstrated highly resolved X‐ray imaging with a high resolution of 15.7 lp/mm and a detection limit as low as 8.8 nGy s^−1^.^[^ ^23^ ^]^

However, most of the investigated Mn‐based halides are focused on the organic‐inorganic hybrid single crystals or micrometer‐sized phosphors.^[^ ^19^, ^21^, ^22^ ^]^ Even though KMnF_3_ perovskite nanocrystals have been synthesized in glasses,^[^ ^24^ ^]^ all‐inorganic cesium manganese halide NCs and their quantum confinement effects are still lack of investigation. In addition, these cesium manganese halide NCs are highly sensitive to oxygen and moisture, which leads to structural transformation and reduction in PL QY.^[^ ^20^, ^25^ ^]^ Therefore, it is highly urgent to find proper ways to encapsulate these cesium manganese halide NCs. As a common synthetic method, in‐situ precipitation of oxide, fluoride, sulfide, selenide, and cesium lead halide NCs into the inorganic glass has been reported in many previous works.^[^ ^7^, ^26^, ^27^, ^28^, ^29^ ^]^ Especially for CsPbX_3_ NCs in glass, their stabilities against air and water have been effectively enhanced via encapsulation by the dense glass network and are much better than colloidal NCs.^[^ ^7^ ^]^ In addition, in situ precipitation of CsPbX_3_ NCs in large‐area glass can be directly applied for LED and X‐ray imaging without postsynthetic encapsulation. Based on mentioned‐above advantages, in‐situ precipitation of lead‐free cesium manganese halide in glass not only yields long‐term stability afforded by the glass matrix, but also maintains their optical performance such as high PL, QY, and large Stokes shift.

In this work, using the conventional melt‐quenching and subsequent thermal treatment method in Scheme S1, Supporting Information, in situ precipitation of lead‐free CsMnCl_3_ NCs, CsMnBr_3_ NCs, and Cs_3_MnI_5_ NCs in glasses are realized for the first time. These cesium manganese halide NCs embedded glasses not only exhibit strong red PL with a relatively high PL QY, but also good thermal and chemical stabilities. Most importantly, these cesium manganese halide NCs embedded glasses are highly photoluminescent and stable under X‐ray irradiation, making them promising as scintillator for X‐ray imaging.

## 2 Results and Discussion

**Figure 1:**
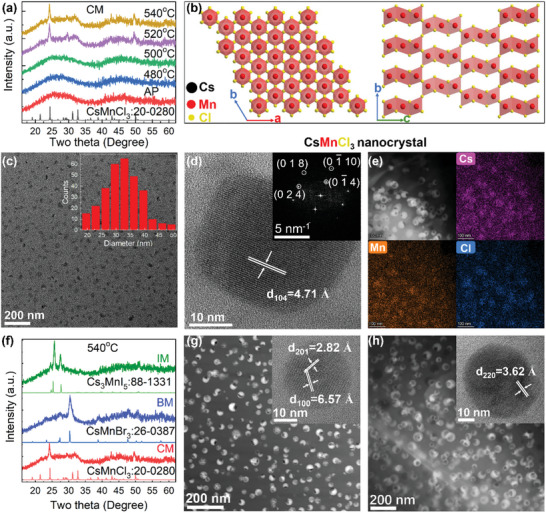
Structural characterization of cesium manganese halide NCs embedded glasses. a) XRD patterns of CM glass specimens heat‐treated at different temperatures. AP represents as‐prepared specimen. b) Schematic illustration of structure of CsMnCl_3_ crystal. c) TEM image of CM specimen heat‐treated at 540 °C for 10 h, and the inset is the size distribution of NCs shown in the image. d) HR‐TEM image one nanocrystal shown in (c), and the inset is corresponding FFT pattern. e) High angle annular dark field image of CM specimen heat‐treated at 540 °C for 10 h and its elemental mapping of Cs, Mn, and Cl. f) XRD patterns of CM, BM, and IM specimens heat‐treated at 540 °C for 10 h. High angle annular dark field TEM image and HR‐TEM (inset) images of g) BM and h) IM specimens heat‐treated at 540 °C for 10 h.

Using melt‐quenching and subsequent thermal treatment, cesium manganese halide NCs are precipitated in glasses (see details in Section 4 and Scheme 1, Supporting Information). For the as‐prepared specimen in chloride glass (named as CM), only a broad diffraction halo is observed, indicating that the as‐prepared CM glass specimen is mainly amorphous and no detectable crystalline phases are present (**Figure** **1**a). When the as‐prepared specimens are heat‐treated at 520 or 540 °C, several diffraction peaks corresponding to CsMnCl_3_ crystal with hexagonal structure (PDF# 20–0280) appear in the diffraction patterns, indicating that hexagonal structured CsMnCl_3_ nanocrystals are precipitated in these heat‐treated CM specimens. For the as‐prepared specimens heat‐treated at 480 and 500 °C, no such diffraction peaks are observed, probably due to the small size and low volume concentration of these nanocrystals precipitated in the glass. In hexagonal‐structured CsMnCl_3_, each manganese ions are coordinated with six Cl^−^ ions to form the octahedron [MnCl_6_]^4−^ units, which are connected through corner‐ and face‐sharing to form long chain (Figure 1b). Precipitation of the CsMnCl_3_ nanocrystals in the heat‐treated CM specimen is further confirmed using HR‐TEM images and elemental mapping (Figure 1c–e). Figure 1c shows the TEM image recorded from CM specimen heat‐treated at 540 °C for 10 h. Nanocrystals are homogeneously precipitated in the glass matrix (Figure 1c), and the average diameter of these nanocrystals are found to be ≈34 nm (inset in Figure 1c). HR‐TEM of one nanocrystal reveals the clear lattice fringe (Figure 1d), indicating good crystalline quality of the nanocrystals formed in the glass. The lattice spacing (*d*_104_ = 4.71Å) and the fast Fourier transformation (FFT) pattern (inset in Figure 1d) are consistent with the hexagonal structured CsMnCl_3_ crystal. HR‐TEM images of more nanocrystals (Figure S1, Supporting Information) further confirm the formation of CsMnCl_3_ NCs in CM specimen. Unlike the Mn: CsPbX_3_ MCs,^[^ ^30^, ^31^ ^]^ no planar defects are observed in these CsMnCl_3_ NCs precipitated in glasses, however, these CsMnCl_3_ NCs can be damaged by electron beam (Figure S1b–e, Supporting Information). Elemental mapping results show that nanocrystals are rich in Cs, Mn, and Cl (Figure 1e), and deficient in B (Figure S2a, Supporting Information), Al (Figure S2b, Supporting Information), Ca (Figure S2c, Supporting Information), Na (Figure S2d, Supporting Information), Sn (Figure S2e, Supporting Information), and O (Figure S2f, Supporting Information). These results demonstrate that NCs formed in CM specimens are CsMnCl_3_ NCs.

Change in the composition of the glass leads to precipitation of cesium manganese bromide NCs in bromide glass (BM) and cesium manganese iodide NCs in iodide glass (IM). Diffraction peaks observed from heat‐treated BM specimens belong to the hexagonal‐structure CsMnBr_3_ crystal (PDF#26‐0387) (Figure S3a, Supporting Information and Figure 1f). CsMnBr_3_ NCs precipitated in BM specimen show similar spherical shape (Figure 1g) and the average diameter of these NCs are found to be ≈33 nm (Figure S4a, Supporting Information). Interplanar distances of 6.57 and 2.82 Å are close to the (100) and (201) lattice spacings of hexagonal‐structure CsMnBr_3_ crystal (Figure 1g). The FFT pattern (Figure S4b, Supporting Information) of one nanocrystal formed in BM specimen also shows that these nanocrystals have hexagonal structure. Elemental mapping of CsMnBr_3_ NCs in Figure S5, Supporting Information shows that these NCs are rich in Cs, Mn, and Br, confirming the formation of CsMnBr_3_ NCs in heat‐treated BM specimens. For heat‐treated IM specimens, diffraction peaks recorded are consistent with tetragonal structured Cs_3_MnI_5_ crystal (PDF#88‐1331, Figure 1f and Figure S3b, Supporting Information), and NCs with average diameter of ≈52 nm (Figure S6a, Supporting Information) are also homogeneously distributed in the glass matrix (Figure 1h). HR‐TEM image of one nanocrystal (inset in Figure 1h), FFT pattern (Figure S6b, Supporting Information), and elemental mapping results (Figure S7, Supporting Information) all confirm that NCs formed in heat‐treated IM specimen are tetragonal‐structured Cs_3_MnI_5_ NCs. It has to be pointed out that black spots observed in the TEM images (Figure 1e,g,h) indicate that these cesium manganese halide NCs can also be destroyed by electron irradiation, which is similar those observed from cesium lead halide nanocrystals.^[^ ^7^, ^30^, ^31^ ^]^ These results show that through adjusting the halide compositions, CsMnCl_3_ NCs, CsMnBr_3_ NCs, and Cs_3_MnI_5_ NCs are precipitated in CM specimen, BM specimen, and IM specimen, respectively.

**Figure 2:**
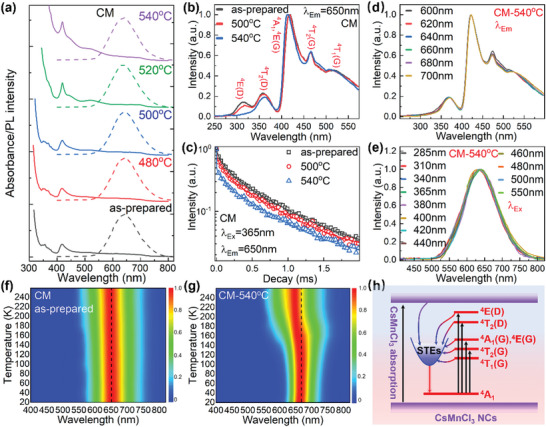
Optical properties and local structure analysis of CsMnCl_3_ NCs in glass. a) Absorption (solid line) and PL (dashed line) spectra of as‐prepared (AP) and heat‐treated CM specimens. b) PLE spectra of as‐prepare CM specimen and CM specimens heat‐treated at 500 and 540 °C for 10 h. c) PL decay curves of as‐prepare CM specimen and CM specimens heat‐treated at 500 and 540 °C for 10 h. d) PLE spectra recorded at different monitoring wavelength and e) PL spectra recorded at different excitation wavelength of CM specimen heat‐treated at 540 °C for 10 h. Low‐temperature (20—260 K) PL spectra of f) as‐prepared CM specimen and g) CM specimen heat‐treated at 540 °C for 10 h recorded using 365 nm light as excitation. h) Schematic diagram of the PL mechanism of CsMnCl_3_ NCs in CM specimens.

The optical properties of CsMnCl_3_ NCs embedded CM glass are investigated by UV–vis absorption, PL excitation (PLE), PL, and time‐resolved PL (TRPL) spectra. As shown in **Figure** **2**a, CM specimens show three absorption peaks at 370, 418, and 523 nm, assigned to the Mn^2+^: ^6^A_1_→^4^E(D) transition, ^6^A_1_→^4^A_1_, ^4^E_1_(G) transition, and ^6^A_1_→^4^T_1_ transition, respectively. As the heat‐treatment temperature increases from 480 to 540 °C, absorption edges of CM specimens exhibit obvious redshift from 315 nm to 373 nm, revealing the formation and growth of CsMnCl_3_ NCs in glass. During heat‐treatment, Mn^2+^ ions participate in the formation of CsMnCl_3_ NCs, and their local environment change from amorphous into crystalline, leading to the enhanced absorption at 418 and 523 nm (Figure 2a). The precipitation of CsMnCl_3_ NCs in glass also has a great effect on the PL properties of CM specimens. For the as‐prepared CM specimen, a broad‐band red PL centering at 644 nm is observed with a full width at half maximum (FWHM) of 108 nm (Figure 2a). When the heat‐treatment temperature increases from 480 to 540 °C, the red PL shifts from 643 to 642, 640, and 637 nm and the FWHM value changes from 108 to 105, 109, and 112 nm, respectively (Figure 2a). As well known, the ^4^T_1_(G)→^6^A_1_(S) transition of tetrahedrally coordinated Mn^2+^ ions gives green PL and octahedrally coordinated Mn^2+^ ions yields red PL.^[^ ^17^ ^]^ Apparently, red PL of the as‐prepared and heat‐treated CM specimens observed in Figure 2a are both from Mn^2+^ ions in octahedral coordination. In order to distinguish the origin of red PL of CM specimens, PLE spectra monitored at red PL are recorded (Figure 2b). When monitored at 650 nm, five typical PLE bands of CM specimens at 315, 359, 419, 467, and 518 nm are observed (Figure 2b), corresponding to the ^6^A_1_→^4^E(D), ^6^A_1_→^4^T_2_(D), ^6^A_1_→^4^A_1_,^4^E(G), ^6^A_1_→^4^T_2_(G) and ^6^A_1_→^4^T_1_(G) transitions of Mn^2+^ ions in octahedral coordination, respectively.^[^ ^20^, ^25^ ^]^ These five PLE bands indicate that the red PL of the as‐prepared and heat‐treated CM specimens stem from the ^4^T_1_(G)→^6^A_1_(S) transition of octahedrally coordinated Mn^2+^ ions. While, with the increase in heat‐treatment temperature, transitions of ^6^A_1_→^4^E(D), ^6^A_1_→^4^T_2_(D) and ^6^A_1_→^4^A_1_,^4^E(G) exhibit slight redshift (Figure 2b). In the meantime, the relative PLE intensities of ^6^A_1_→^4^E(D) and ^6^A_1_→^4^T_2_(D) transitions gradually decrease. When the heat‐treatment temperature is higher than 500 °C, the PLE band due to ^6^A_1_→^4^E(D) transition is nearly smoothed out (Figure 2b). These changes show that part of the Mn^2+^ ions in the glass matrix are participated into CsMnCl_3_ NCs in CM specimens during heat‐treatment. These differences are more clearly demonstrated in the low‐temperature PLE spectra recorded from the as‐prepared and heat‐treated specimens (Figure S7, Supporting Information). With the precipitation of CsMnCl_3_ NCs in glass, PL decay curves of the CM specimens also changes significantly (Figure 2c). The average lifetime of the red PL decreases from 460.5 to 417.5 and 370.3 µs for as‐prepared specimen and those heat‐treated at 500 and 540 °C (Figure 2c). In addition, these PL decay curves can be fitted using a bi‐exponential function, and the fitting results are shown in Figure S9, Supporting Information. It can be found that the slow decay time constant is in the range of 376–499 µs, comparable to those observed from CsMnCl_3_ and CsMnBr_3_ single crystal.^[^ ^20^, ^32^ ^]^ The fast decay time constant decreases from 67.9 µs (as‐prepared specimen) to 36.5 µs (500 °C) and 3.5 µs (540 °C) (Figure S9a, Supporting Information), attributed to the gradual formation of Mn–Mn pairs.^[^ ^33^, ^34^ ^]^ With the increase in heat‐treatment temperature, the proportion of the fast decay process increases from 38% to 62% (Figure S9b, Supporting Information), resulting in the shortening of the average lifetimes. In hexagonal CsMnCl_3_ crystal, the small Mn‐Mn distance of 4.1 Å makes an important contribution to quench the PL from Mn^2+^ ions. Thus, the PL QYs of CM specimens increase from 18.9% (AP specimen) to 41.8% (480 °C), and then decrease to 27.5% (500 °C), 19.4% (520 °C), 11.7% (540 °C). It is also noted that PLE spectra (Figure 2d) and PL spectra (Figure 2e) are almost independent on the monitoring wavelength and excitation wavelength, demonstrating that red PL of CsMnCl_3_ NCs comes from the same excited state of Mn^2+^ ions.

For cesium metal halides with soft lattices such as Cs_2_ZnX_4_, CsAgX_2_, and Cs_3_Cu_2_X_5_, the strong electron–phonon interaction can cause transient lattice distortion and formation of self‐trapped excitons (STEs), leading to broad band PL with large Stokes shift.^[^ ^35^, ^36^ ^]^ To give the direct evidence for the formation of self‐trapping exciton in cesium manganese halide NCs, the femtosecond transient absorption spectra of as‐prepared and heat‐treated specimens are shown in Figure S10, Supporting Information. For as‐prepared specimen, there is no signal observed in the transient absorption spectra (Figure S10a, Supporting Information). Once CsMnCl_3_ NCs are precipitated into CM glass, the Pseudo color transient‐absorption of CM heated specimens possesses a broad photo‐induced absorption (PIA) band at the range of 500≈810 nm (Figure S10b, Supporting Information), which matches well the red PL of CsMnCl_3_ NCs. This positive absorption signal is similar to the absorption signal of STE in halide double‐perovskite NCs^[^ ^35^, ^36^ ^]^, and confirms that red PL of CsMnCl_3_ NCs originates from STE, instead of the d–d transition of Mn^2+^ ions. In order to illustrate the effect of electron–phonon interaction on the optical properties of CsMnCl_3_ NCs, low‐temperature (20–260 K) PL spectra of as‐prepared CM specimen and CM specimen heat‐treated at 540 °C are recorded using 365 nm light as excitation. For the as‐prepared CM specimen, peak wavelength and FWHM of PL bands remain nearly constant (Figure 2f). While, for heat‐treated CM specimen, PL band exhibits no shift when the temperature is lower 100 K, and blue‐shifts from 665 to 644 nm with further increase in temperature (Figure 2g). FWHM of the PL band from heat‐treated CM specimen gradually broadens from 63.5 nm to 110.4 nm with increase in temperature (Figure 2g). Using the Equation (S1), Supporting Information, Huang‐Rhys factor (S) and phonon frequency ($\hbar \omega_{\mathrm{phonon}}$) are calculated to be 39.2 and 10.5 meV (Figure S11a, Supporting Information). The obtained S factor is comparable to those of double‐perovskite NCs (30–80),^[^ ^35^, ^36^, ^37^ ^]^ and much larger than many semiconductors (0.3–5) and Mn^2+^ ions doped II‐VI QDs (2.3–3.5).^[^ ^38^, ^39^ ^]^ Using the Toyozawa equation (Equation (S2), Supporting Information), the electron–phonon coupling energy (Γ_*op*_) is found to be 193 meV (Figure S11b, Supporting Information), similar to Cs_2_ZnCl_4_ NCs (Γ_*op*_= 215 meV).^[^ ^40^ ^]^ Using the Equation (S3), Supporting Information, exciton binding energy (*E_b_*) is estimated to be 59.4 meV (Figure S11c, Supporting Information). For as‐prepared CM specimen, the S factor (Figure S11d, Supporting Information), electron–phonon coupling energy (Γ_*op*_) (Figure S11e, Supporting Information), and exciton binding energy (Figure S11f, Supporting Information) are calculated to be 6.68, 82, and 19.6 meV, respectively. Interestingly, the low‐temperature PL spectra recorded from CsMnCl_3_ NCs embedded CM specimen using 460 nm light as excitation also give S, Γ_*op*_, and *E_b_* values of 12.2, 198, and 55 meV, respectively (Figure S12, Supporting Information), and the low‐temperature PL spectra recorded using 525 nm light as excitation give S, Γ_*op*_, and *E_b_* values of 9.7, 136, and 32.6 meV, respectively (Figure S13, Supporting Information). It is found that the Huang‐Rhys factor S (9.7) recorded at 525 nm light excitation is much bigger than that of the d‐d transition of Mn^2+^ ions in glass matrix (6.68) in Figure S11d, Supporting Information, demonstrating that red PL upon 525 nm light excitation still performs strong electron‐phonon coupling. As a result, the transient absorption, the large S factor, and large electron‐phonon coupling energy obtained from CsMnCl_3_ NCs embedded CM specimen demonstrate that sub‐bandgap excitation of Mn^2+^ ions can also induce the formation of STEs and generation of red PL. The PL mechanism diagram of CsMnCl_3_ NCs could be described in Figure 2h. After the photoexcitation, electron–phonon interactions are strong enough for excited electrons and holes to cause transient lattice distortion and further lead to the formation of STE, resulting in the STE PL with broad band width and large Stokes shift through the radiative recombination between STE states and ^6^A_1_ state of Mn^2+^ ions.

**Figure 3:**
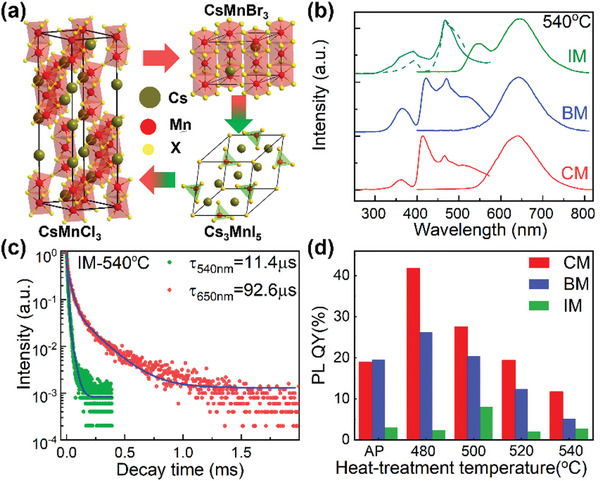
Spectroscopic characterization of cesium manganese halide NCs embedded glasses. a) Schematic structures of CsMnCl_3_, CsMnBr_3_, and Cs_3_MnI_5_ crystals. b) Comparison of PLE and PL spectra of CM, BM, and IM specimens heat‐treated at 540 °C for 10 h. PL spectra are recorded using 365 nm light as excitation, and PLE spectra are recorded by monitoring the PL at 650 nm for CM and BM specimens. PLE spectra of IM specimen are recorded at 540 nm (dashed lines) and 650 nm (solid line). c) PL decay curves recorded at 540 nm (green points) and 650 nm (red points) from IM specimen heat‐treated at 540 °C for 10 h. The blue lines represent the biexponential function fitting. f) PL QYs of CM, BM, and IM specimens heat‐treated at different temperatures. AP represents as‐prepared specimen.

As illustrated above, substitution of halides results in the formation of hexagonal CsMnBr_3_ NCs in BM specimens and tetragonal Cs_3_MnI_5_ NCs in IM specimens (**Figure** **3**a). Compared with the absorption spectra recorded from CM specimens (Figure 2a), absorption edges of heat‐treated BM specimens (Figure S14a, Supporting Information) and IM specimens (Figure S15a, Supporting Information) appear at longer wavelength side when these specimens are thermally treated using the same temperature and duration (Figure S17, Supporting Information). Such red‐shifts in absorption edges are mainly induced by the reduction in effective band gap energies of CsMnBr_3_ NCs and Cs_3_MnI_5_ NCs. Since CsMnCl_3_ NCs and CsMnBr_3_ NCs have the same hexagonal structure, PL spectra (Figure S14b, Supporting Information), PLE spectra (Figure S14c, Supporting Information), and PL decay curves (Figure S14d, Supporting Information) of BM specimens are similar to those observed from CM specimens (Figure 2). Compared with CsMnCl_3_ NCs and CsMnBr_3_ NCs, Cs_3_MnI_5_ NCs precipitated in IM specimens have tetragonal structure and exhibit different optical properties (Figure 3b,c, and Figure S15, Supporting Information). For the as‐prepared IM specimen, only one broad PL band centering at 652 nm is observed (Figure S15b, Supporting Information), which is from the octahedrally coordinated Mn^2+^ ions in glass matrix. When Cs_3_MnI_5_ NCs are precipitated in IM specimens upon heat‐treatment, another green PL band located at 546 nm with a FWHM of 51 nm is observed (Figure 3b), and PL intensity of this green PL band increases with the increase in heat‐treatment temperature (Figure S15b, Supporting Information). PLE spectra recorded by monitoring the green and red PL of heat‐treated IM specimens are also largely different from those recorded from the as‐prepared IM specimen (Figure S15c, Supporting Information), CM specimens (Figure 2b), and BM specimens (Figure S14c, Supporting Information). Compared with the long decay lifetime of red PL (100–200 µs) (Figure S15d, Supporting Information and Figure 3c), the PL decay lifetimes of the green band recorded from the heat‐treated IM specimens are found to be in the order of ≈10 µs (Figure 3c and Figure S16, Supporting Information). These results demonstrate that the green PL at 546 nm of heat‐treated IM specimens is from the tetragonally coordinated Mn^2+^ ions in Cs_3_MnI_5_ NCs, instead from the octahedrally coordinated Mn^2+^ ions in glass matrix. Low‐temperature PL spectra are also recorded from the CsMnBr_3_ NCs in BM specimen heat‐treated at 540 °C (Figure S18, Supporting Information) and Cs_3_MnI_5_ NCs in IM specimen heat‐treated at 540 °C (Figure S19, Supporting Information). Using the Equations. (S1)–(S3), Supporting Information, the S factors, electron‐phonon coupling energy (Γ_*op*_), and exciton binding energy (*E_b_*) are found to be 11.1, 314, and 27.9 meV for CsMnBr_3_ NCs in BM specimen (Figure S18, Supporting Information), and 18.4, 11.3, and 41.8 meV for the green band PL from Cs_3_MnI_5_ NCs in IM specimen (Figure S19, Supporting Information). With the precipitation of cesium manganese halide NCs in glass specimens, the PL QYs of these NCs embedded specimens are improved, and the maximal PL QYs for CsMnCl_3_ NCs embedded CM specimens, CsMnBr_3_ embedded BM specimens, and Cs_3_MnI_5_ NCs embedded IM specimens are 41.8%, 26.2%, and 8%, respectively (Figure 3d).

**Figure 4:**
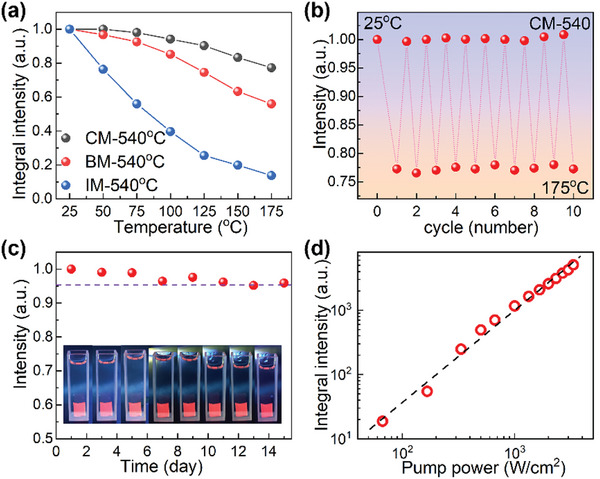
Long‐term stability of cesium manganese halide NCs embedded glasses. a) Temperature‐dependent PL intensities of heat‐treated CM, BM, and IM specimens. All these specimens are excited by 365 nm light. b) Thermal‐cycling (25–175 °C) induced changes in PL intensity of heat‐treated CM specimen. c) Water‐immersion induced changes in PL intensity of heat‐treated CM specimen. Inset are images of heat‐treated CM specimen immersed in water recorded under 365 nm light illumination. d) Dependence of PL intensity of heat‐treated CM specimen on excitation power density of 365 nm light. All these specimens are heat‐treated at 540 °C for 10 h.

Even though cesium manganese halide NCs are sensitive to moisture, oxygen, and high energy photon, incorporation of these NCs into glass matrix can improve their stabilities. With the increase in temperature, PL intensities of these NCs embedded specimens decrease (**Figure** **4**a and Figure S20, Supporting Information). At 100 °C, these NCs embedded CM, BM, and IM specimens maintain 94.1%, 85.2%, and 39.7% of their PL intensities recorded at room temperature, and at 175 °C, these NCs embedded CM, BM, and IM specimens maintain 77.2%, 55.9%, and 13.7% of their PL intensities recorded at room temperature (Figure 4a and Figure S20, Supporting Information). For CsMnCl_3_ NCs embedded CM specimen, the integrated PL intensity can be almost completely recovered after ten heat‐cooling cycles (Figure 4b), and CsMnBr_3_ NCs embedded BM specimen and Cs_3_MnI_5_ NCs embedded IM specimen also show similar results during heat‐cooling processing (Figure S21, Supporting Information). These results show that these cesium manganese halide NCs embedded glasses have relatively good thermal stabilities. In addition, incorporation of these cesium manganese halide NCs into glasses can greatly improve their chemical stabilities, since the dense and inert glass matrix provide protection of these NCs from the harsh environment. For example, for CM specimen heat‐treated at 540 °C, its PL intensity exhibits only ≈4% reduction after immersing in water for 15 days (Figure 4c). Besides the good thermal and chemical stabilities, these cesium manganese halide NCs embedded glasses are also resistant to intense UV light irradiation. For CsMnCl_3_ NCs embedded CM glass, their PL intensities monotonically increase when the excitation power density (at 365 nm) increases from 66.7 to 3333.3 W cm^−2^ (Figure 4d and Figure S22, Supporting Information), and no obvious changes in spectral lineshape in PL spectra and no visible light‐induced damages on the specimen is observed (Figure S22, Supporting Information). All these results verify that these cesium manganese halide NCs embedded glass specimens possess good thermal‐, chemical‐, and photo‐stabilities, promising for practical applications.

**Figure 5:**
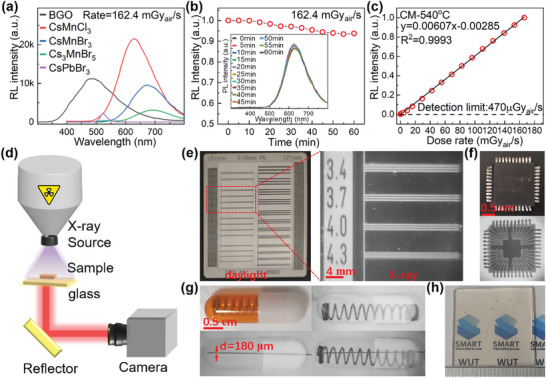
Scintillator performance of cesium manganese halide NCs embedded glasses. a) RL spectra of CM, BM, and IM specimens heat‐treated at 540 °C for 10 h, BGO single crystal and CsPbBr_3_ NCs embedded glass under X‐ray excitation with a dose rate of 162.4 mGy_air_ s^−1^. b) Changes in RL intensity of CM specimen heat‐treated at 540 °C upon continuous irradiation by X‐ray with a dose rate of 162.4 mGy_air_ s^−1^. c) Dependence of RL intensity of CM specimen heat‐treated at 540 °C on X‐ray irradiation dose rate. d) Schematic illustration of the in‐direct X‐ray imaging system. Photographs and X‐ray (dose rate of 20 mGy_air_ s^−1^) images of e) standard X‐ray resolution pattern plate, f) one chip, and g) one capsule with spring and needle inside, recorded with CM specimen heat‐treated at 540 °C for 10 h. h) Heat‐treated CM specimen with a size of 3 cm × 3 cm × 2 mm for X‐ray imaging.

Considering the relative high PL QY, good stability, lead‐free, and reduced reabsorption, these cesium manganese halide NCs embedded glasses have great potential for luminous scintillator (Scheme S2, Supporting Information). To demonstrate their potential, radioluminescence (RL) properties of CsMnCl_3_ NCs embedded glass, CsMnBr_3_ NCs embedded glass, and Cs_3_MnI_5_ NCs embedded glass are compared with CsPbBr_3_ NCs embedded glass (Supplementary Note 1) and Bi_4_Ge_3_O_12_ (BGO) single crystal, and all these specimens are shaped into same sizes. As shown in **Figure** **5**a, RL intensities recorded from cesium manganese halide NCs embedded specimens are much larger than that recorded from CsPbBr_3_ NCs embedded specimen, and RL intensities recorded from CsMnBr_3_ NCs embedded BM specimen and CsMnCl_3_ NCs embedded CM specimen are comparable to even higher than that recorded from BGO single crystal, indicating that these cesium manganese halide NCs embedded glass specimens can convert X‐ray to visible photons efficiently. Since BGO single crystal has a known light yield about 8600 ph MeV^−1^,^[^ ^41^, ^42^ ^]0^ the light yield of CsMnCl_3_ NCs embedded glass is estimated about 13 400 ph MeV^−1^ according to the Equation (S4), Supporting Information (Note S3, Supporting Information). Similarly, the light yield of CsMnBr_3_ NCs, Cs_3_MnBr_5_ NCs, and CsPbBr_3_ NCs embedded glassed are estimated to 5800, 1900, and 340 ph MeV^−1^, respectively. These cesium manganese halide NCs embedded glasses are also highly resistant to X‐ray irradiation. For CsMnCl_3_ NCs embedded specimen, 94% of the initial RL is remained after exposure to X‐ray with a dose rate of 162.4 mGy_air_ s^−1^ for 60 min (Figure 5b), which is better than CsPbBr_3_ NCs embedded glass (≈90% remained upon 8 mGy_air_/s X‐ray irradiation for 5 h)^[^ ^43^ ^]^ and CsMnCl_3_: 1% Pb NCs (90% remained upon 18 mGy_air_ s^−1^ X‐ray irradiation for 60 min).^[^ ^44^ ^]^ The XRD pattern in Figure S23a, Supporting Information indicates no structural change in CsMnCl_3_ NCs appears after the X‐ray irradiation. In addition, the X‐ray RL intensity of the CsMnCl_3_ NCs embedded specimen also shows a linear dependence on the irradiation dose rate when it increases from 0.5 to 162.4 mGy_air_ s^−1^ (Figure S23b, Supporting Information and Figure 5c). This linear dependence of integrated RL intensity on the incident X‐ray dose rate is beneficial for achieving a good‐X‐ray imaging contrast. Using a linear fitting, the minimal detectable dose rate of X‐ray is found to be 470 µGy_air_ s^−1^ for CsMnCl_3_ NCs embedded specimen. As a proof‐of‐concept, an X‐ray imaging system consisting of X‐ray tube and digital camera is used to study the X‐ray imaging ability of CsMnCl_3_ NCs embedded glass scintillator with a size of 3 cm×3 cm×2 mm (Figure 5d,h). As shown in Figure 5e, a standard test‐pattern plate (0.1 mm Pb) is used to estimate the spatial resolution of CsMnCl_3_ NCs embedded glass scintillator. The glass scintillator achieves the spatial resolution of 4.0 lp mm^−1^, which is comparable to the resolution of CsPbBr_3_ QDs embedded glass (4.1 lp mm^−1^)^[^ ^13^ ^]^ and CsMnCl_3_: 1% Pb NCs film (4.3 lp mm^−1^).^[^ ^44^ ^]^ To further illustrate the potential towards X‐ray imaging, one chip (Figure 5f) and one capsule (Figure 5g) are imaged using the CsMnCl_3_ NCs embedded CM specimen. Under X‐ray irradiation with a dose rate of 20 mGy_air_ s^−1^, the threadlet in the chip (Figure 5f) and the outline of fine needle or spring in the capsule (Figure 5g) can be clearly observed. Especially, the needle with a diameter of 180 µm in the complex structure is clearly demonstrated (Figure 5g). These X‐ray imaging results show that these cesium manganese halide NCs, especially CsMnCl_3_ NCs embedded glass can be used as scintillator of X‐ray imaging application.

## 3 Conclusions

In summary, lead‐free cesium manganese halide NCs (including CsMnCl_3_, CsMnBr_3_ and Cs_3_MnI_5_) embedded glasses are successfully prepared for the first time through a simple melt‐quenching and subsequent heat‐treatment. Highly efficient red PL with broad PL band and PL QY as high as 41.8% is obtained. In addition, under different halogen components, the coordination environment of Mn^2+^ ions in NCs is modulated from the octahedral coordination in CsMnCl_3_ and CsMnBr_3_ NCs to the tetrahedral coordination in Cs_3_MnI_5_ NCs, leading to adjustable PL region from red PL to green PL. It is found that CsMnX_3_ NCs embedded glass exhibits excellent long‐term stability. On account of negligible self‐absorption, long‐term stability and high PL QY, CsMnX_3_ NCs embedded glass achieves highly‐efficient and stabile RL, presenting excellent X‐ray scintillation performance and highly resolved X‐ray imaging. This work promotes the development of high‐performance, stabile and lead‐free metal halide materials for X‐ray scintillation.

## 4 Experimental Section

Cesium manganese halide NCs embedded glasses were prepared using the conventional melt‐quenching and subsequent thermal treatment method. Nominal compositions of the glass were 40B_2_O_3_‐19Al_2_O_3_‐10CaO‐5Cs_2_O‐15NaX‐10MnX_2_‐1SnO (in mol%), where X = Cl, Br, I. SnO was introduced to generate the reducing environment to maintain the divalent state of Mn ions. Glasses containing chloride, bromide, and iodide were named as CM, BM, and IM, respectively. No glass with mixed halides were prepared in this work. Raw materials were mixed thoroughly and then melted in alumina crucibles at 1300 °C for 40 min under ambient atmosphere. After melting, glass melts were poured onto preheated brass mold for quenching, and the quenched glasses were further annealed at 350 °C for 2 h to reduce the thermal stress built during the quenching process. Glasses thus obtained were named as as‐prepared glasses. Cesium manganese halide NCs were precipitated in glasses via thermal treatment at temperatures ranging from 480 to 540 °C for 10 h. Based on the types of the halide compounds, CsMnCl_3_ NCs, CsMnBr_3_ NCs, and Cs_3_MnI_5_ NCs were formed in the glass matrices after thermal treatment. No cesium manganese halide NCs with mixed halides embedded glasses were prepared in this work.

X‐ray diffraction patterns of the as‐prepared glass specimens and heat‐treated specimens were recorded with X‐ray diffractometer (XRD, D8 Advance, Germany). Microstructures and compositions of the precipitated NCs in glasses were analyzed using high‐resolution transmission electron microscope (HR‐TEM, Talos‐F200s, USA) equipped with energy‐dispersive X‐ray spectroscopic (EDS) analyzer. Specimens for TEM characterization were prepared using focused ion beam (Helios Nanolab DualBeam, Thermo Scientific, USA) and the thickness of these specimens were thinned down to <60 nm to reduce the charging effect. Absorption spectra of the as‐prepared glasses and heat‐treated specimens were recorded using an UV/vis/NIR spectrophotometer (Lambda 750s, PerkinElmer, USA). PL and PL excitation (PLE) spectra of as‐prepared glasses and heat‐treated specimens were recorded using a time‐resolved fluorescence spectrometer (FL3‐22, Jobin‐Yvon, USA). Low‐temperature PL and PLE spectra were recorded using a close‐cycled cryostat system cooled by compressed helium gas (Optistat AC‐V12, Oxford, UK). The absolute PL QYs of specimens containing cesium manganese halide NCs were measured using a UV–NIR quantum yield spectrometer (C13534, Quantaurus‐QY Plus, Hamamatsu, Japan). The µs‐scale PL decay curves were measured using one spectrofluorometer (FSL 1000, Edinburg, UK) with an Xe lamp as the excitation source. Radioluminescence (RL) spectra were collected by an Omni‐300i spectrofluorometer (Zolix, China) equipped with an X‐ray tube (50 kV, maximal power 12 W, Cu target). The X‐ray images were recorded using a digital camera (Canon 5D4) under 50 kV X‐ray irradiation.

## Conflict of Interest

The authors declare no conflict of interest.

## Supporting information

Supporting information

## Data Availability

The data that support the findings of this study are available in the supplementary material of this article.
