# Supplementary material for: Lead‐Free Cesium Manganese Halide Nanocrystals Embedded Glasses for X‐Ray Imaging
Source: Adv Sci (Weinh). 2022 Dec 3;10(4):2204843. doi: 10.1002/advs.202204843 (PMC9896042; doi:10.1002/advs.202204843)
Supplement: Supplementary file 1 — Supporting information [file ADVS-10-2204843-s001.pdf]

ADVANCED SCIENCE
Open Access

Supporting Information

for *Adv. Sci.*, DOI 10.1002/advs.202204843

Lead-Free Cesium Manganese Halide Nanocrystals Embedded Glasses for X-Ray Imaging

*Kai Li, Wenchao Zhang, Luyue Niu, Ying Ye, Jing Ren and Chao Liu**

Supporting Information

Lead-free cesium manganese halide nanocrystals embedded glasses for X-ray imaging

Kai Li[1], Wenchao Zhang[1], Luyue Niu[2], Ying Ye[1], Jing Ren[2], Chao Liu[1,*]

[1]State Key Laboratory of Silicate Materials for Architectures (SMART), Wuhan University of Technology, 122 Luoshi Road, Hongshan, Wuhan, Hubei 430070, P. R. China

[2]Key Laboratory of In-fiber Integrated Optics, Ministry Education of China, Harbin Engineering University, Harbin 150001, China

E-mail：hite@whut.edu.cn

Supplementary Note 1:

To evaluate the scintillator performance of cesium manganese halide NCs embedded glass, $CsPbBr_3$ NCs embedded glass is selected as control sample. $CsPbBr_3$ NCs are precipitated into borosilicate glass with a nominal composition (mol%) of $39SiO_2$-$39B_2O_3$-5ZnO-5CaO-$5Cs_2O$-2PbO-25NaBr. The detailed preparation process is described in our previous work [1]. $CsPbBr_3$ NCs embedded glass with a high PL QY of 80% is obtained through thermal treatments at temperatures of 500 °C for 2 h.

Supplementary Note 2:

Temperature-dependent PL line broadening is fitted by Huang-Rhys factor (S) using the following equation [2]:

$$FWHM(T) = 2.36\sqrt{S}\hbar\omega_{phonon}\sqrt{coth\frac{\hbar\omega_{phonon}}{2k_BT}} \qquad \text{(S1)}$$

Where $\hbar$ is reduced Planck constant, ω_{phonon} is the phonon frequency, T is the temperature, and k_B is the Boltzmann constant. Fig. S9a, S9d, S10b, S15b, S16b, and S16e are fitted using Eq. S1.

Temperature-dependent PL line broadening in Fig. S9b and S9d is fitted using Toyozawa equation [3]:

$$\Gamma(T) = \Gamma_0 + \Gamma_{op}/\left(e^{\hbar\omega_{op}/k_bT} - 1\right) \qquad \text{(S2)}$$

Where Γ_0 is the intrinsic line width at 0 K, Γ_{op} is the electron-phonon coupling energy, $\hbar\omega_{op}$ is the longitudinal optical phonon energy and k_B is the Boltzmann constant. Fig. S9b, S9e, S10c, S15c, S16c, and S16f are fitted using Eq. S2.

Temperature-dependent PL intensities in Fig, S9c and S9f is fitted using the following equation [4]:

$$I(T) = \frac{I_0}{1+Aexp(-\frac{E_b}{k_BT})} \qquad \text{(S3)}$$

where I_0 is the intensity at 0 K, E_b is the exciton binding energy, k_B is the Boltzmann constant. Fig. S9c, S9f, S10d, S15d, S16d, and S16g are fitted using Eq. S3.

Supplementary Note 3:

In order to estimate the light yield of $CsMnCl_3$ NCs embedded glass, $Bi_4Ge_3O_{12}$ (BGO) single crystal is used as a reference, which have a similar size and a known light yield about 8600 ph/MeV [5,6], according to the equation

$$\frac{LY_{CsMnCl_3}}{LY_{BGO}} = \frac{R_{BGO}}{R_{CsMnCl_3}} \times \frac{\int I_{CsMnCl_3}(\lambda)d\lambda \times S_{CsMnCl_3}}{\int I_{BGO}(\lambda)d\lambda \times S_{BGO}} \qquad \text{(S4)}$$

where R is the X-ray deposited energy percentage of scintillators, I is the radioluminescence intensity at different wavelengths (λ), and S is the irradiation area. Due to the same sample thickness (about 2 mm, most of the X-ray photons are absorbed) at the same test condition, the light yield of $CsMnCl_3$ NCs is estimated to 13400 ph/MeV according to the integrated intensity, which is 1.56 of BGO single crystal. Similarly, the light yield of $CsMnBr_3$ NCs, Cs_3MnBr_5 NCs and $CsPbBr_3$ NCs embedded glass are estimated to 5800 ph/MeV, 1900 ph/MeV and 340 ph/MeV according to the integrated intensity, which is 0.68, 0.23 and 0.04 of BGO single crystal, respectively.

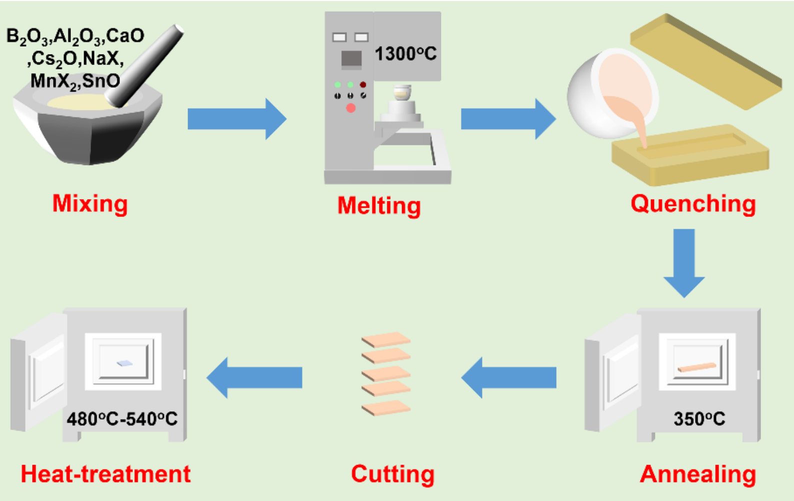

Scheme S1. Schematic illustration on the preparation of cesium manganese halide NCs embedded glass.

"This synthesis process is conventional and similar to the procedure to make glass-ceramics containing nanocrystals. This synthesis method is well documented in literatures and has been widely used to precipitate semiconductor nanocrystals in

glass, since the first discovery of semiconductor nanocrystals with quantum confinement effect [7]. Using this method, many types of semiconductor nanocrystals have been precipitated in glasses, including CdX [8], ZnX [9], PbX (X=S, Se, Te) [10], cesium lead halide [11], and cesium zinc halide [12] nanocrystals or quantum dots.

To precipitate semiconductor nanocrystals, the following synthesis steps are involved.

(1) Preparation of the raw chemical powders: Design the glass composition is the key point to precipitate certain type of semiconductor nanocrystals in glass, since type, structure, and composition of the semiconductor nanocrystals, and whether it can be precipitated or not in glass is determined by the composition and structure of the glass. When the composition of the glass is properly designed, it is possible to make many types of semiconductor nanocrystals in glass. According to the designed composition of glass, choose proper chemicals, weight, and mix thoroughly to prepare homogeneous chemical powders. Ball milling and grinding are usually used for the thorough mixing of the weighted raw chemical powders.

(2) Melting to prepare the precursor glass: After mixing, put the mixed chemical powders into crucible, and then put them into high temperature furnace. Temperature of the furnace used to melt the glass is determined by the composition of the glass, and one can find the rough temperature for melting through empirical equation calculation and optimize the melting temperature through several trials. One needs to adjust the melting temperature and duration to make sure that all chemical powders are melted and homogenized during the melting process. After melting, the raw chemical powders become glass melt, and all the constituent elements of the raw chemicals are present in the glass melt either in form of structural units such as [SiO_4] and [BO_3] to form the amorphous network of glass, or ionic state such as Cs^+ ions as structural modifiers to maintain the charge neutrality.

(3) Quenching to form glass: After melting, the crucible is taken out of the furnace and the glass melt in the crucible is poured onto pre-heated mold (normally metallic mold, such as brass mold with high thermal conductivity) to cool down the melt and

form glass. Cooling speed should be controlled in order to get clear glass without precipitating any crystalline phases in the glass. In most cases, viscosity of the glass melt rapidly increases during cooling, diffusion and structural rearrangement becomes more and more difficult for the structural units and structural modifiers in glass melts during cooling process. When the temperature decreases down to glass transition temperature or lower, the glass melt becomes amorphous solid. At this condition, constituent elements of semiconductor are solidified in the amorphous network of glass.

(4) Annealing: Annealing is indispensable step to make glass. During quenching process, the glass suffers large temperature change, and thermal stress is present. Too high thermal stress can easily lead to cracking or even fragmentation of the glass. Annealing of the quenched glass at proper temperature for enough time can largely remove the thermal stress.

(5) Cutting: After annealing, the annealed glass are cut into required shapes for further heat-treatment.

(6) Heat-treatment: Heat-treatment is the step to precipitate semiconductor nanocrystals in glass. Since the constituent elements of semiconductor are solidified in solid and amorphous glass, thermal treatment at elevated temperature (determined from thermal analysis) can drive these elements to diffuse and form nanocrystals inside. Heat-treatment is normally carried out at temperatures higher than the temperature of glass transition. Semiconductor nanocrystals precipitated in the glass is determined by the designed composition of the glass. By adjusting the heat-treatment temperature and duration, size of the nanocrystals can be modulated. Size of the nanocrystals increase with the increase in heat-treatment temperature or prolongation in heat-treatment duration. Thus, by adjusting the thermal treatment condition, band gap energy, absorption, and photoluminescence properties of the precipitated nanocrystals can be tuned.

(7) Polishing: After heat-treatment, the specimen is optically polished for further structural and optical characterization.”

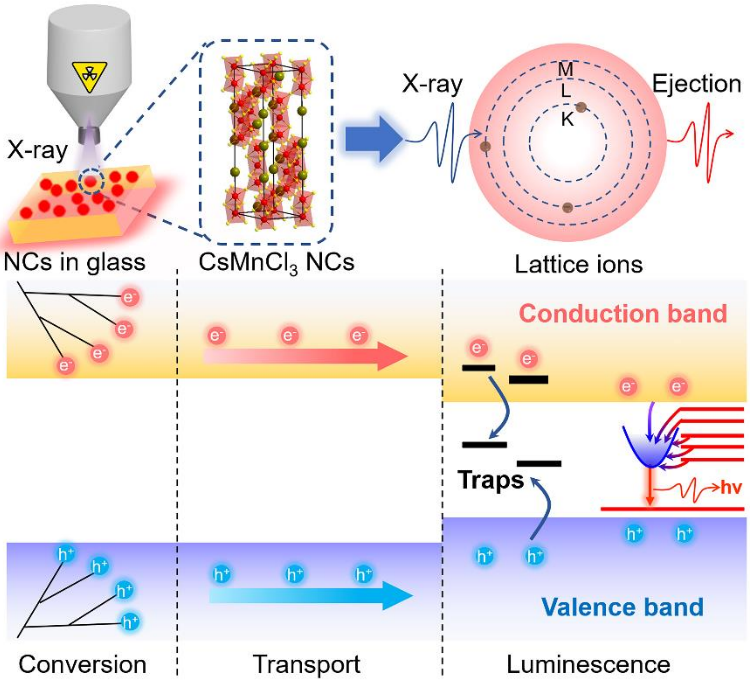

Scheme S2. Schematic illustration of the RL mechanism in $CsMnCl_3$ NCs embedded glass through conversion, transport, and luminescence. Part of the incoming X-ray can interact with heavy atoms (such as Cs^+ and Mn^{2+}), and most of the incoming X-ray is attenuated by the NCs. Due to the high X-ray attenuation ability of these NCs, the lattice atoms of $CsMnCl_3$ NCs can absorb the vast majority of the incoming X-ray radiation to generate hot electrons and deep holes, through the photo-electric effect and inelastic Compton scattering. Subsequently, these charge carriers could thermally dissipate partial energy through interacting with the phonons and gradually accumulate at the conduction band and valence band. These charge carriers at the band edges can recombine radiatively to generate luminescence. These charge carriers can be also trapped by the defects of nanocrystals or transport into glass matrix, leading to the nonradiative recombination.

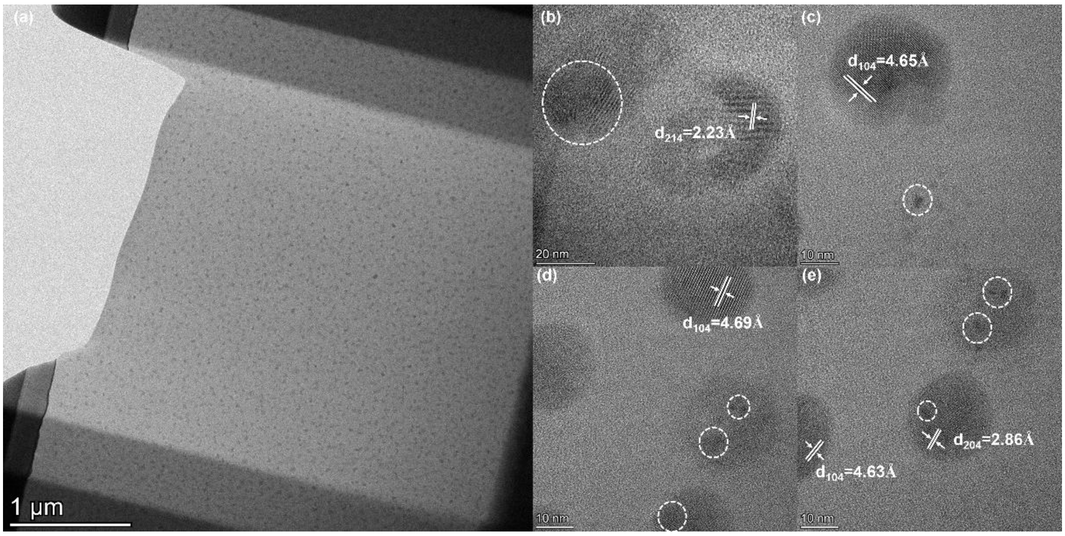

Fig. S1. (a) TEM image of CM specimen prepared using a focused ion beam. (b-e) HR-TEM images of different particles.

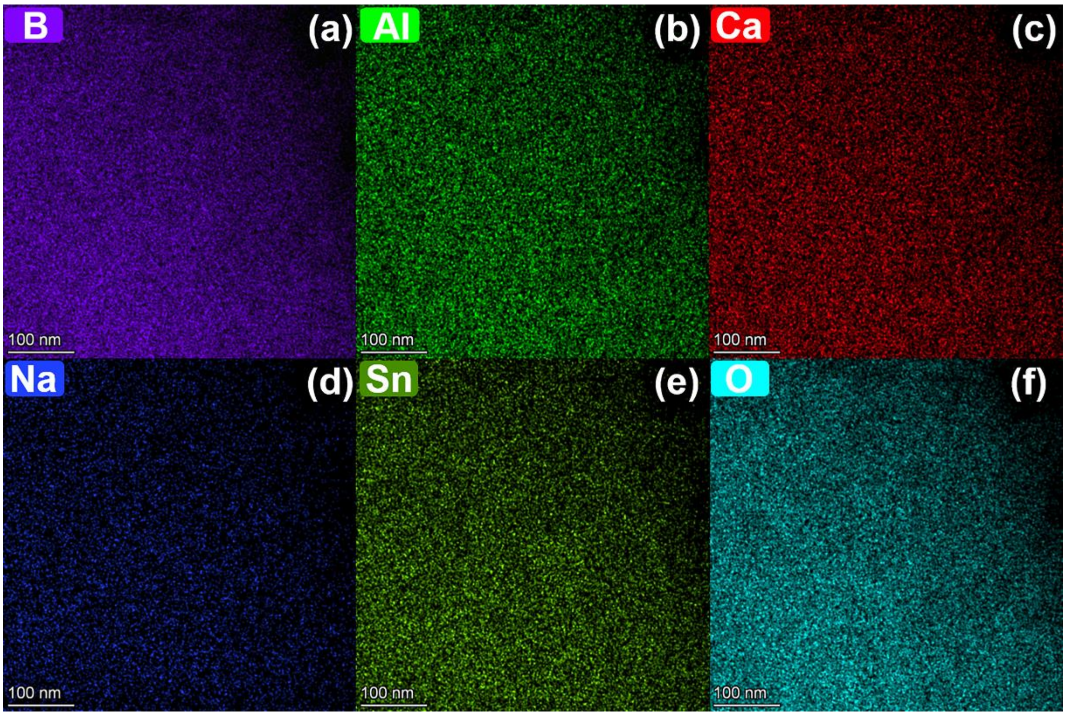

Fig. S2. Element mapping of (a) B, (b) Al, (c) Ca, (d) Na, (e) Sn, and (f) O of CM specimen shown in Fig. 1e.

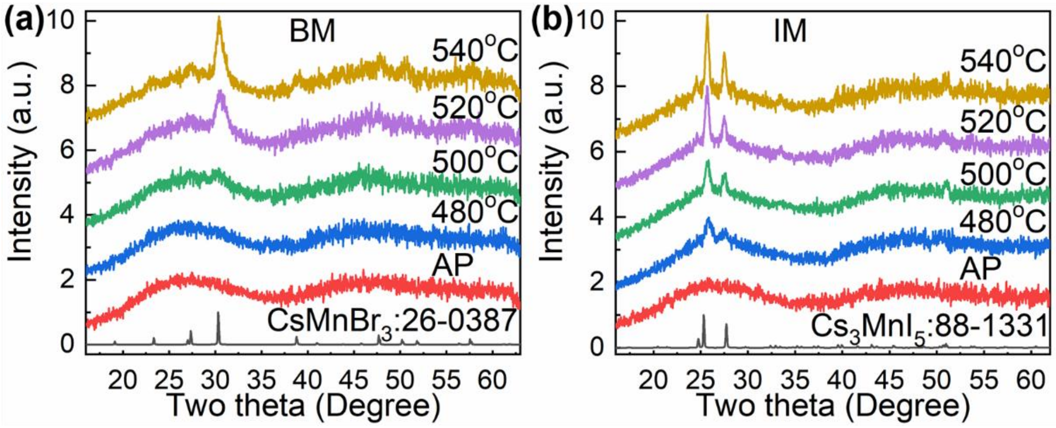

Fig. S3. XRD patterns of (a) BM and (b) IM glass specimens heat-treated at different temperatures for 10 h. AP represents as-prepared specimen.

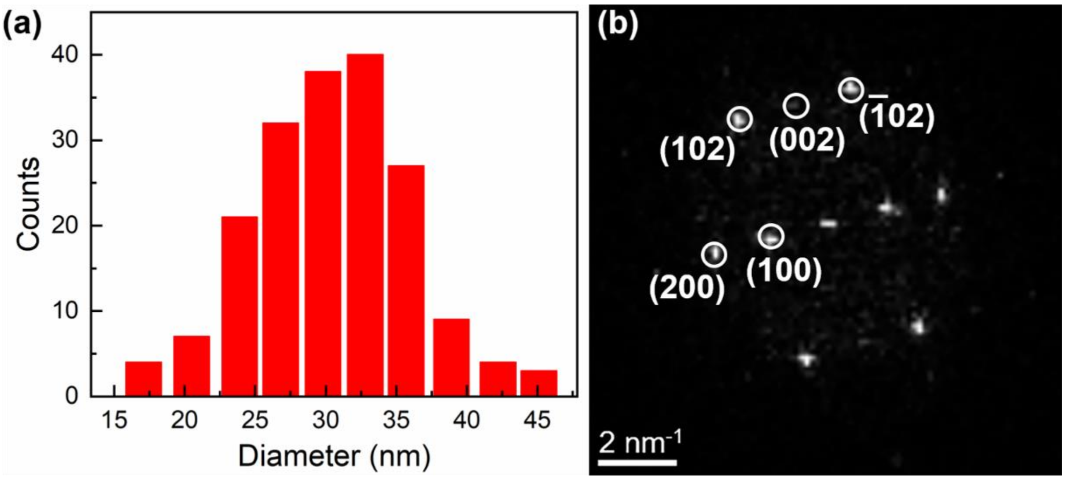

Fig. S4. (a) Size distribution of $CsMnBr_3$ NCs and (b) fast Fourier transformation pattern of one nanocrystal formed in BM specimen heat-treated at 540 ºC for 10 h.

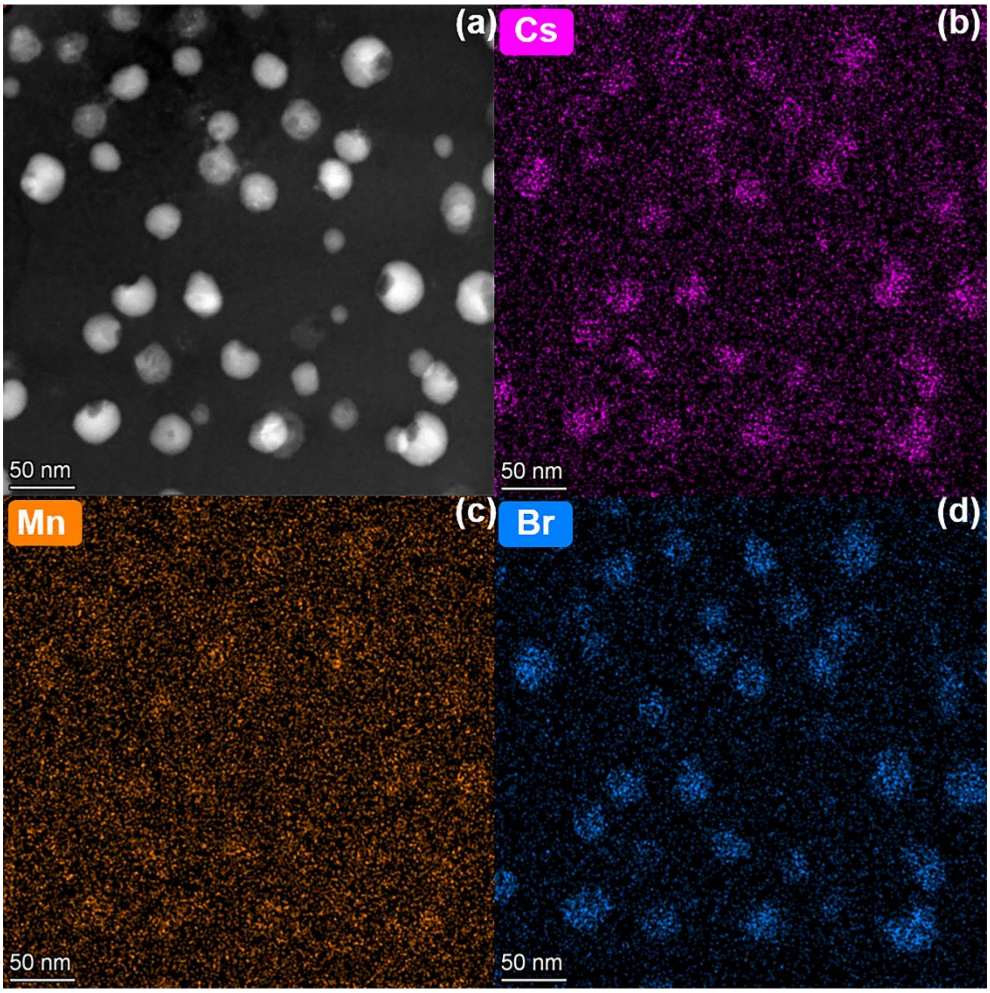

Fig. S5. (a) High angle annular dark field TEM image and corresponding element mapping of (b) Cs, (c) Mn, (d) Br in BM specimen heated at 540 ºC for 10 h.

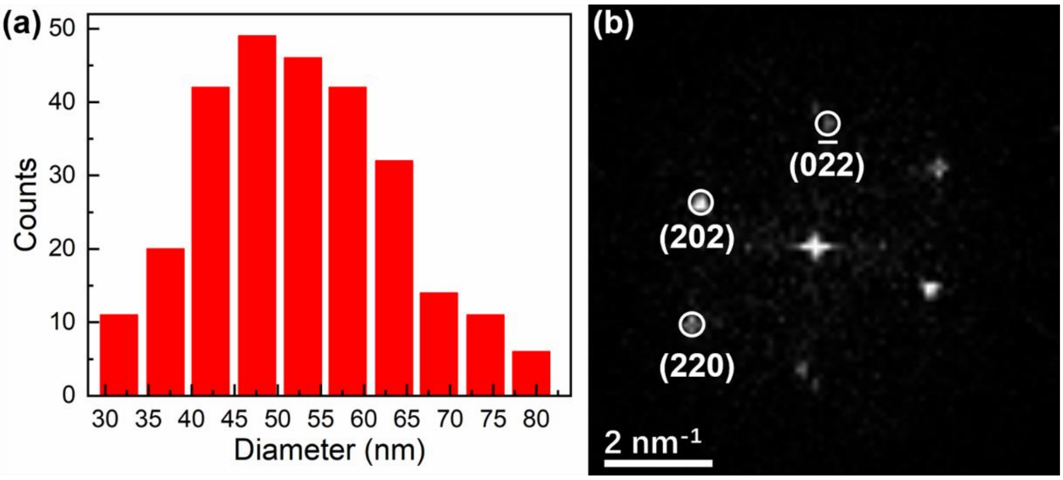

Fig. S6. (a) Size distribution of Cs_3MnI_5 NCs and (b) fast Fourier transformation pattern of one nanocrystal formed in IM specimen heat-treated at 540 ºC for 10 h.

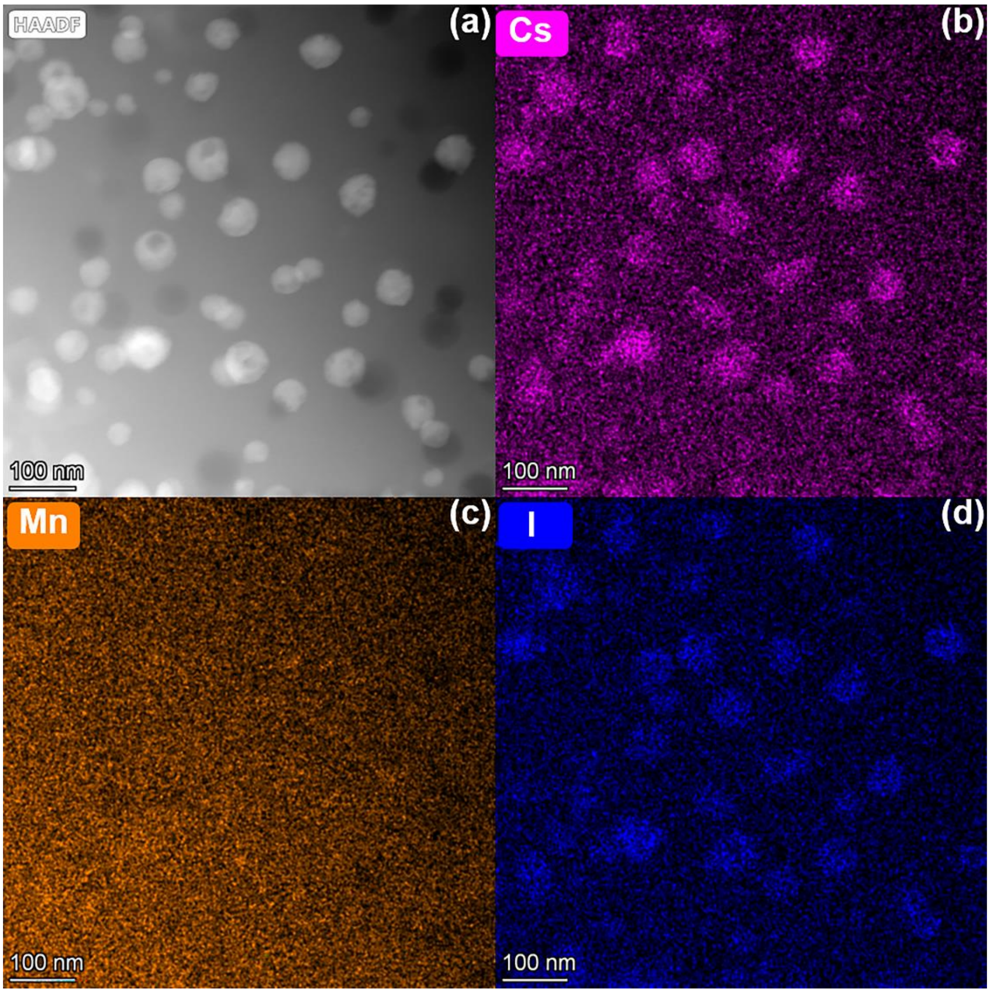

Fig. S7. (a) High angle annular dark field TEM image and corresponding element mapping of (b) Cs, (c) Mn, (d) I in IM specimen heated at 540 ºC for 10 h.

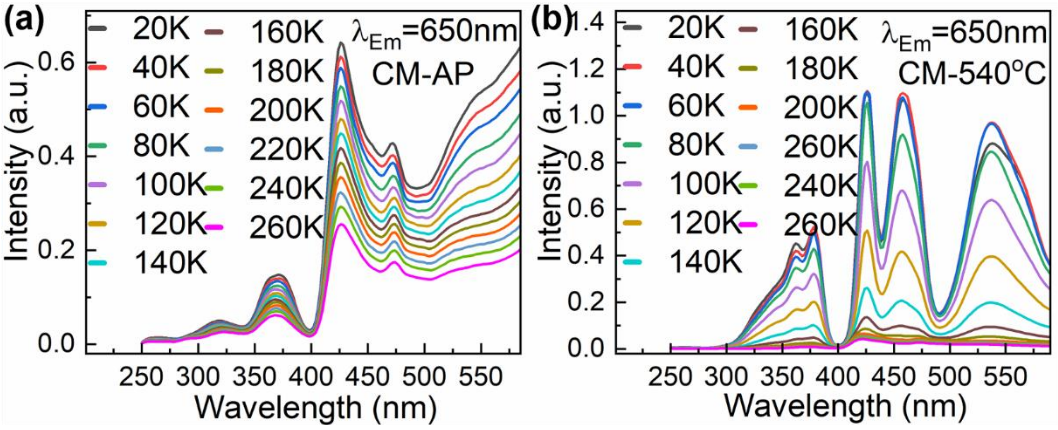

Fig. S8. Low-temperature (20-260K) PLE spectra recorded from (a) as-prepared CM specimen and (b) CM specimen heat-treated at 540 ºC for 10 h. AP represents as-prepared specimen.

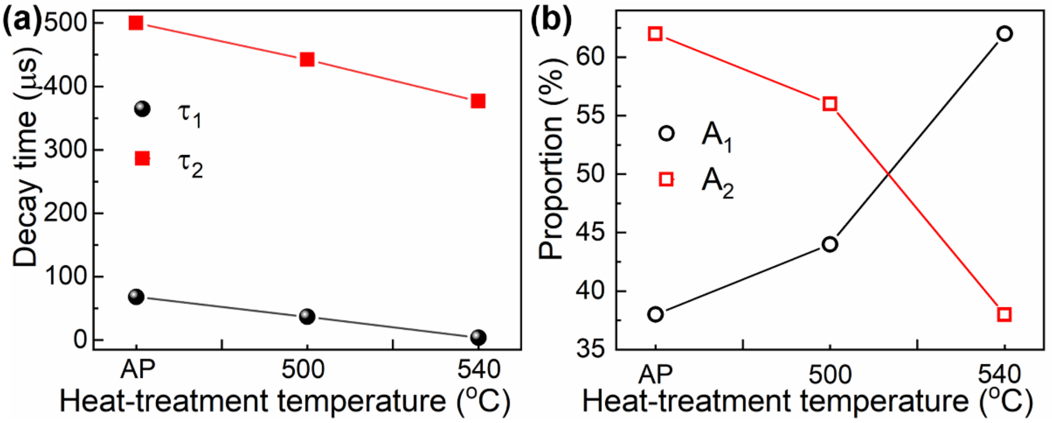

Fig. S9. (a) Fast (τ_1) and slow (τ_2) decay time, (b) proportion of fast (A_1) and slow (A_2) decay component of PL recorded from as-prepared and heat-treated CM specimens.

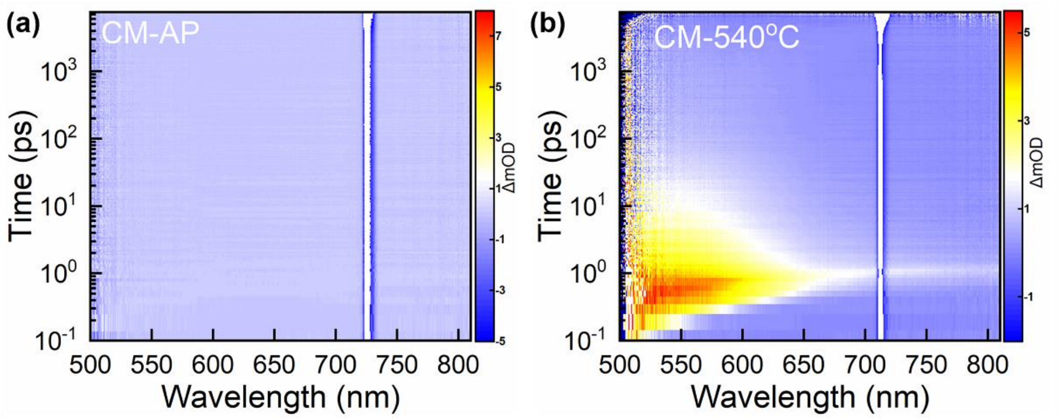

Fig. S10. Femtosecond transient absorption spectra of (a) as-prepared and (b) heat-treated CM specimens recorded under 355 nm laser excitation (40 μJ/cm^2).

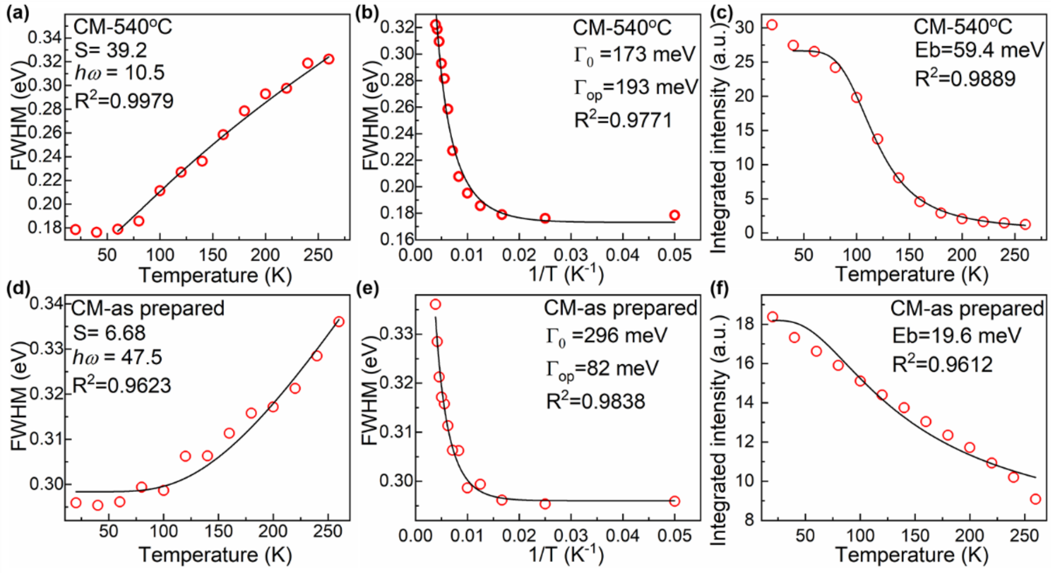

Fig. S11. Fittings of (a,b) FWHM and (c) integrated intensities of low-temperature PL spectra recorded from CM specimen heat-treated at 540 ºC for 10 h (Fig. 2g). Fittings of (d,e) FWHM and (f) integrated intensities of low-temperature PL spectra recorded from as-prepared CM specimen (Fig. 2f).

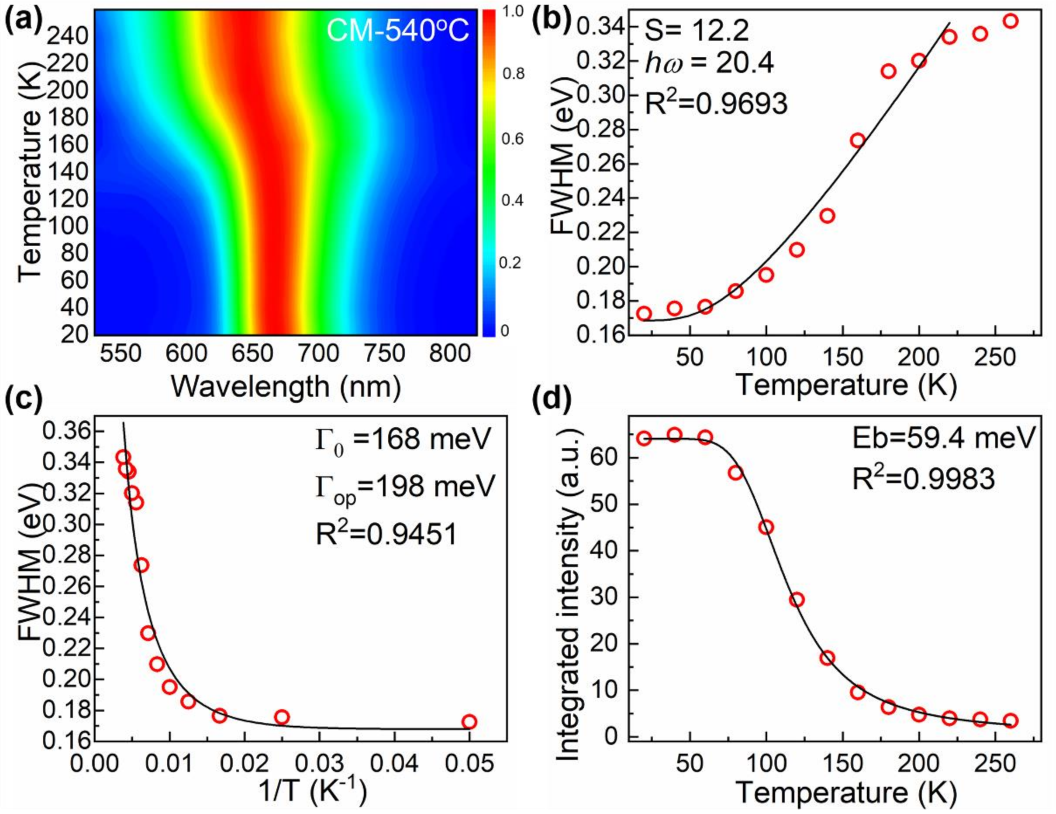

Fig. S12. (a) Low-temperature PL spectra of CM specimen heat-treated at 540 ºC for 10 h, and the PL spectra are recorded using 460 nm light as excitation. Fittings of (b,c)

FWHM and (d) integrated intensities of low-temperature PL shown in (a) using Eq. S1, S2, and S3, respectively.

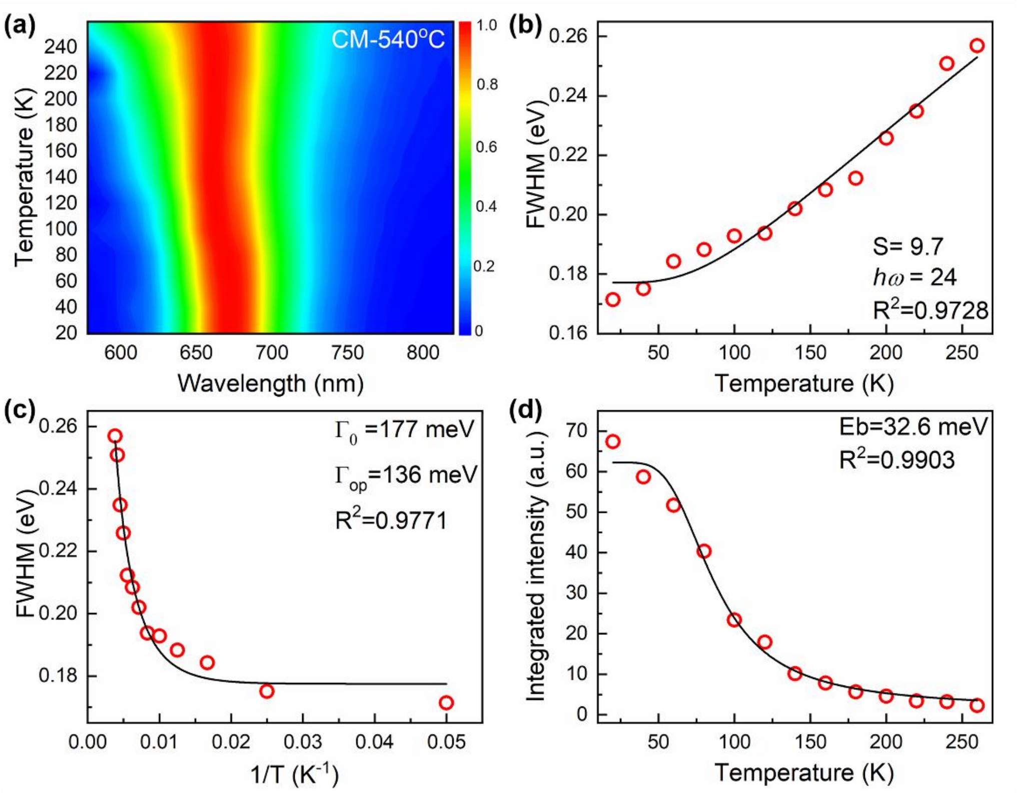

Fig. S13. (a) Low-temperature PL spectra of CM specimen heat-treated at 540 ºC for 10 h, and the PL spectra are recorded using 460 nm light as excitation. Fittings of (b,c) FWHM and (d) integrated intensities of low-temperature PL shown in (a) using Eq. S1, S2, and S3, respectively.

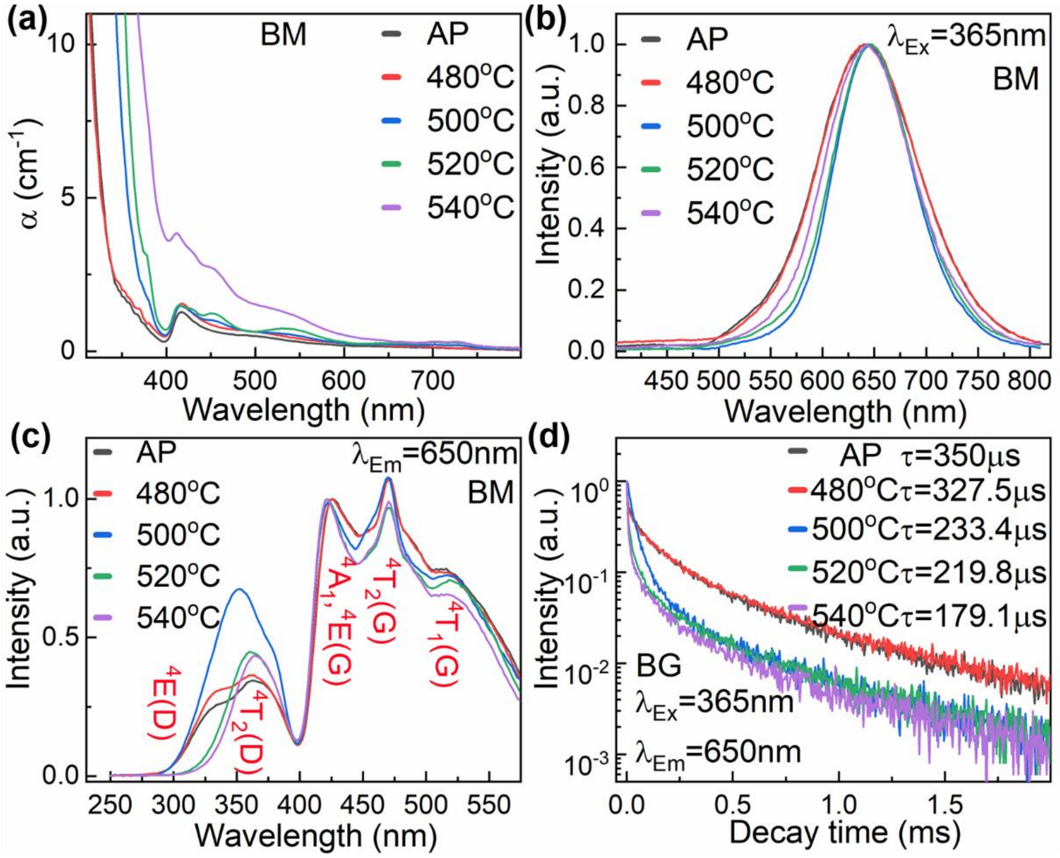

Fig. S14. (a) Absorption spectra, (b) PL spectra, (c) PLE spectra, and (d) photoluminescence decay curves of as-prepared BM specimen and BM specimens heat-treated at different temperatures. AP represents as-prepared specimen.

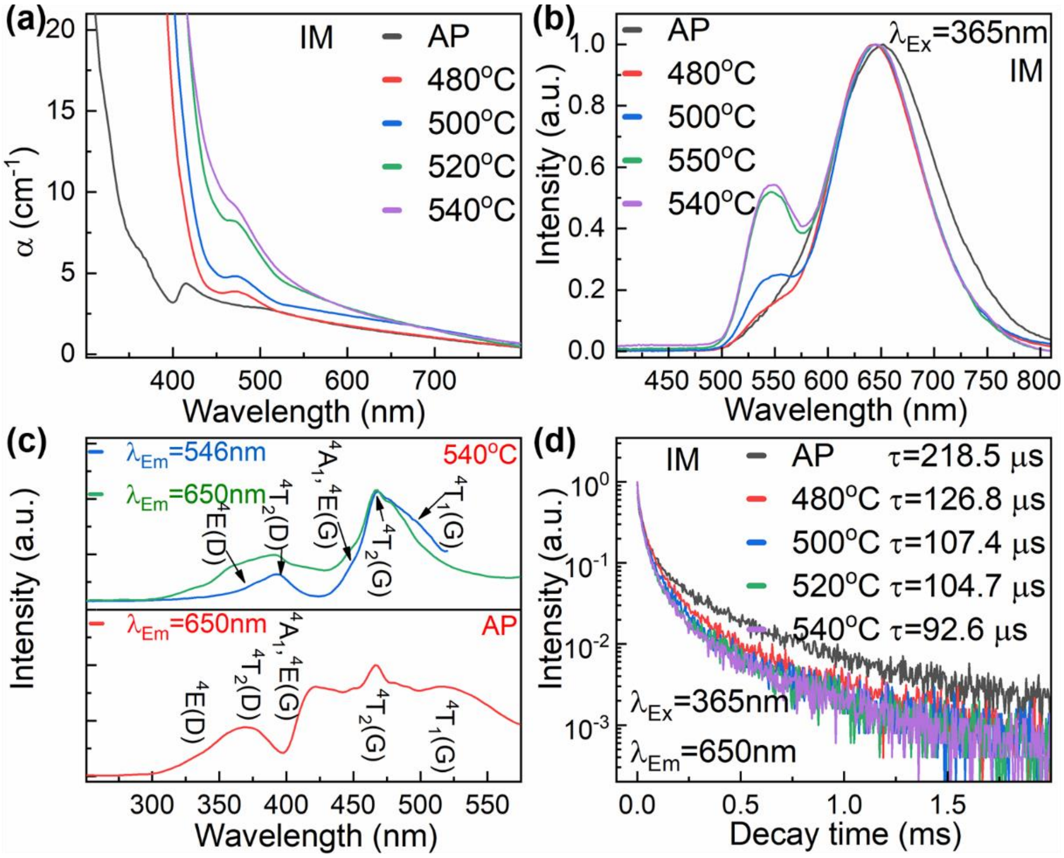

Fig. S15. (a) Absorption spectra, (b) PL spectra, (c) PLE spectra, and (d) photoluminescence decay curves of as-prepared IM specimen and IM specimens heat-treated at different temperatures. AP represents as-prepared specimen.

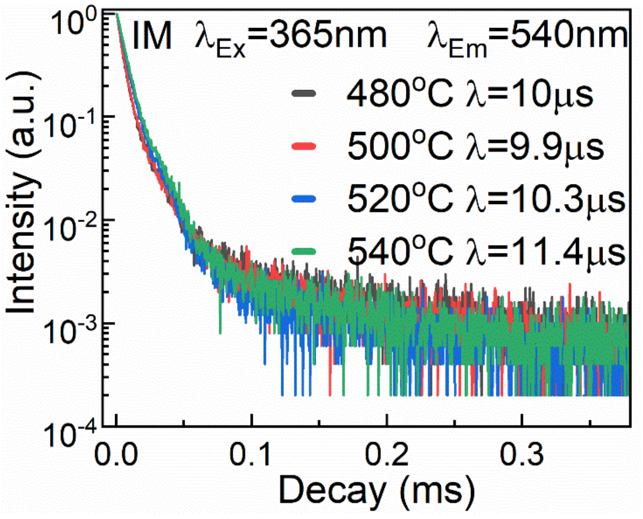

Fig. S16. Photoluminescence decay curves monitored at 540 nm of IM specimens heat-treated at different temperatures.

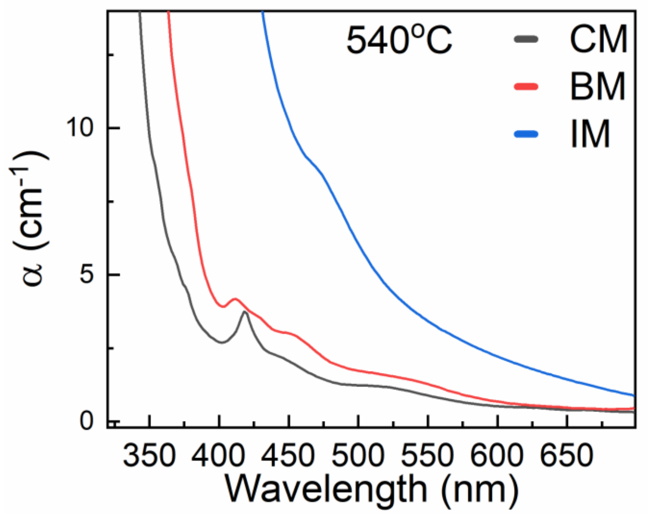

Fig. S17. Comparison of absorption spectra of CM, BM, and IM specimens heat-treated 540 ºC for 10 h.

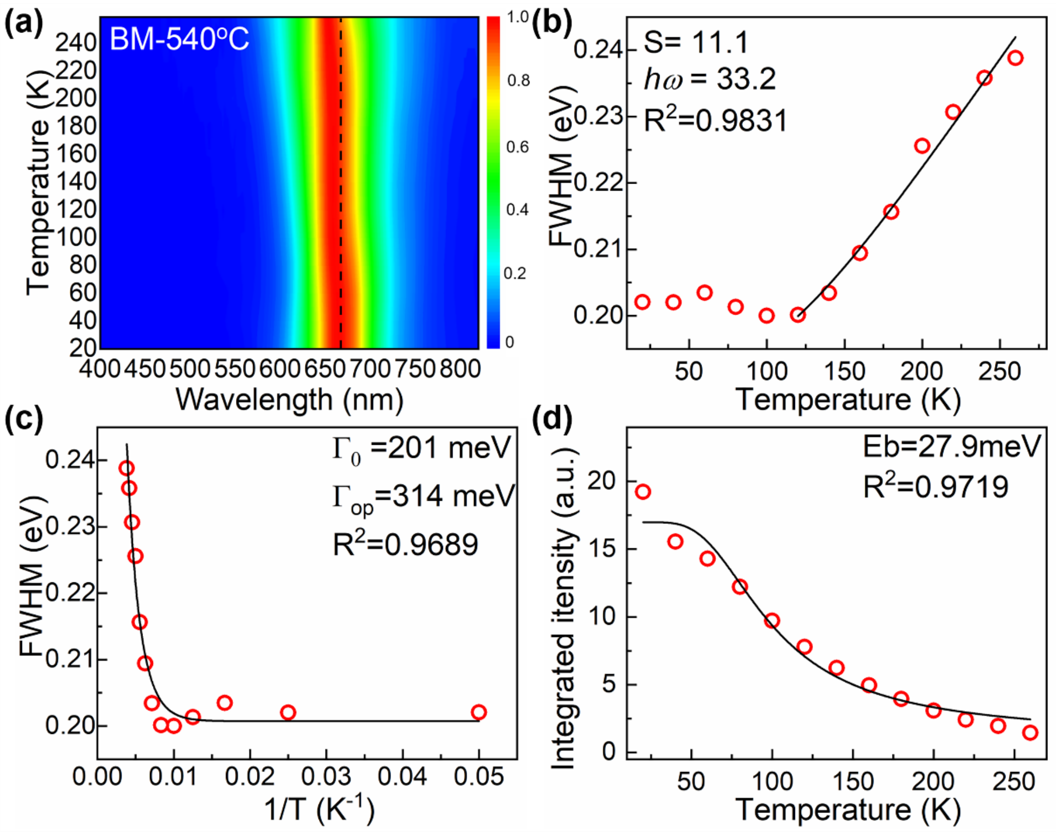

Fig. S18. (a) Low-temperature PL spectra of BM specimen heat-treated at 540 ºC for 10 h, and the PL spectra are recorded using 365 nm light as excitation. Fittings of (b,c) FWHM and (d) integrated intensities of low-temperature PL shown in (a) using Eq. S1, S2, and S3, respectively.

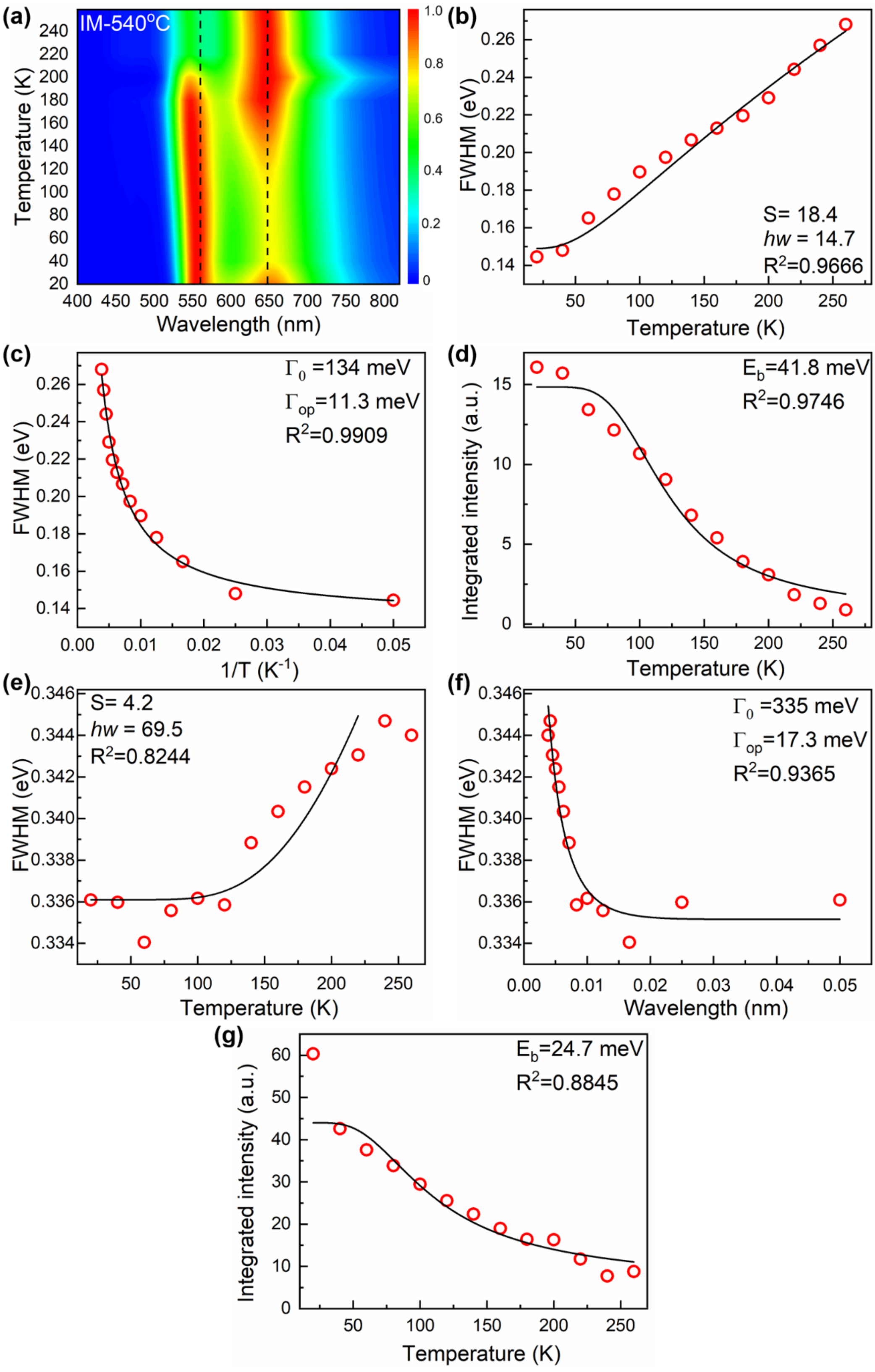

Fig. S19. (a) Low-temperature PL spectra of IM specimen heat-treated at 540 ºC for 10 h, and the PL spectra are recorded using 365 nm light as excitation. Fittings of (b,c) FWHM and (d) integrated intensities of the green PL band shown in (a) using Eq. S1,

S2, and S3, respectively. Fittings of (e,f) FWHM and (g) integrated intensities of the red PL band shown in (a) using Eq. S1, S2, and S3, respectively.

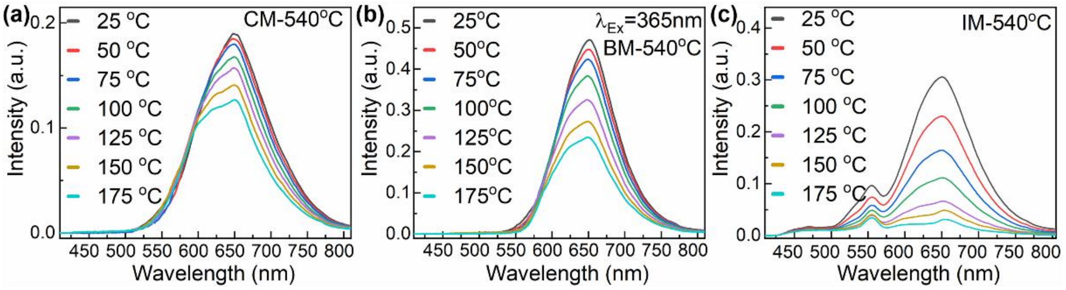

Fig. S20. High-temperature PL spectra of CM, BM, and IM specimens heat-treated 540 ºC for 10 h.

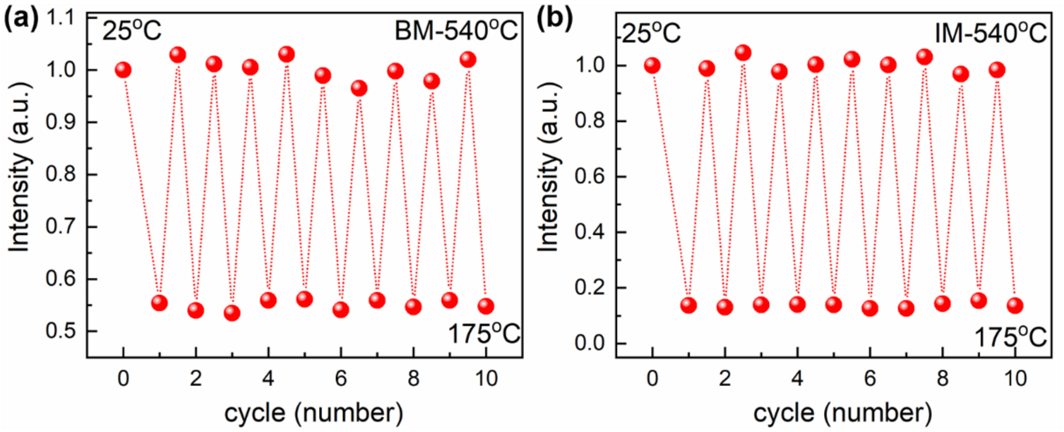

Fig. S21. Thermal-cycling (25-175 ºC) induced changes in PL intensity of (a) BM and (b) IM specimens heat-treated at 540 ºC for 10 h.

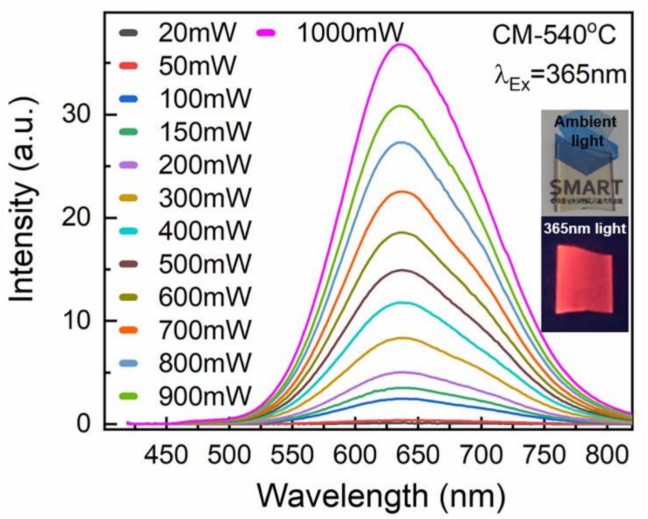

Fig. S22. Effects of excitation power density on the PL spectra of CM specimen heat-treated at 540 ºC for 10 h. The inset shows the images of intense-laser-irradiated CM specimen recorded under ambient light and 365 nm light.

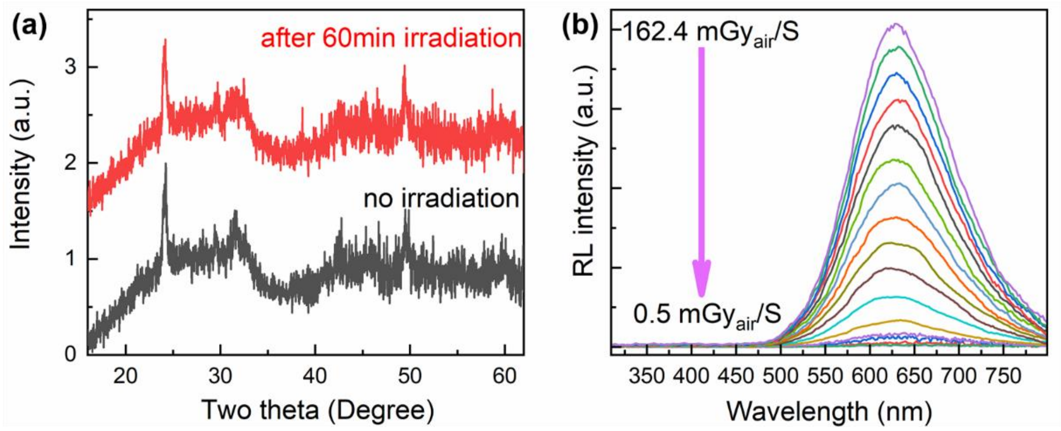

Fig. S23. RL spectra of heat-treated CM specimen (540 ºC for 10 h) recorded at different X-ray irradiation dose rates.

References

[1] Y. Ye, W. C. Zhang, Y. D. Zhang, K. Li, J. J. Han, C. Liu, Chem. Eng. J. 2022, 445, 136867.

[2] W. Stadler, D. M. Hofmann, H. C. Alt, T. Muschik, B. K. Meyer, E. Weigel, G. Müller-Vogt, M. Salk, E. Rupp, K. W. Benz, Phys. Rev. B 1995, 51, 10619.

[3] J. W. Qiao, G. J. Zhou,Y. Y. Zhou, Q. Y. Zhang, Z. G. Xia, Nat. Commun. 2019, 10, 5267.

[4] R. S. Zeng, K. Bai, Q. L. Wei, T. Chang, J. Yan, B. Ke, J. L. Huang, L. S. Wang, W. C. Zhou, S. Cao, J. L. Zhao, B. S. Zou, Nano Res. 2020, 14, 1551.

[5] M. Nikl, Meas. Sci. Technol. 2006, 17, 37.

[6] A. Jana, S. Cho, S. A. Patil, A. Meena, Y. Jo, V. G. Sree, Y. Park, H. Kim, H. Im, R. A. Taylor, Mater. Today 2022, 55,110.

[7] A. I. Ekimov, A. A. Onushenko, V. A. Tzehomskii, Sov Phys Chem Glass 1980, 6, 511.

[8] K. Li, Y. Ye, W. C. Zhang, Y. Z. Hu, Y. Yang, Y. Zhou, C. Liu, J. Mater. Chem. C, 2021, 9, 11261.

[9] K. Li, W. C. Zhang, Y. Ye, C. Liu, Y. Yang, J. Wang, J. Ruan, J. J. Han, Chem. Eng. J. 2021, 410, 128324.

[10] C. Liu, Y. K. Kwon, J. Heo, Appl. Phys. Lett. 2009, 94, 021103.

[11] Y. Ye, W. C. Zhang, Z. Y. Zhao, J. Wang, C. Liu, Z. Deng, X. J. Zhao, Adv. Opt. Mater. 2019, 7, 1801663.

[12] K. Li, Y. Ye, W. C. Zhang, Y. Zhou, Y. D. Zhang, S. S. Lin, H. Lin, J. Ruan, C. Liu, Nano Res. 2022, 15, 9368.
